# Supplementary material for: Fourier-Based 3D Multistage Transformer for Aberration Correction in Multicellular Specimens
Source: ArXiv. 2025 May 23:arXiv:2503.12593v2. Preprint. [Version 2] (PMC12136486)
Supplement: Supplement 1 [file NIHPP2503.12593v2-supplement-1.pdf]

Appendices

APPENDICES

A	Ablation study	16
A.1	Fourier embedding design	16
A.2	Synthetic training and validation data	16
A.3	Multistage design	16
A.4	Training dataset size	17
A.5	Training hyperparameters	17
A.6	Measuring prediction confidence	17
B	Architecture benchmark	18
B.1	Baseline models	18
B.2	Cost analysis	18
C	In-silico evaluations	19
C.1	Sensitivity to SNR	19
C.2	Generalization to other light sheets	19
C.3	Sensitivity to the density of objects	19
C.4	Sensitivity to object size	20
D	Genome editing of ap2s1 for Zebrafish	20

A ABLATION STUDY

A.1 Fourier embedding design

Most ML approaches to AO rely on real-space representations of training data [4–8]. However, open-source AO training datasets remain scarce, and acquiring large, annotated 3D volumes that capture diverse biological features over a broad aberration space is prohibitively expensive. Training on small datasets leads to overfitting and poor generalization; scaling existing 2D models to 3D also comes at significant computational cost.

To address these challenges, we use synthetic data transformed to the Fourier domain. We embed diffraction-limited puncta (such as signal from sub-diffractive beads or fluorescent protein labeled AP2) into Fourier space using a pre-processing step (see Sec. 4.7). Within an isoplanatic patch, the aberration affects signal from all emitters, yielding a consistent Fourier 'fingerprint'. As shown in Supplementary Fig. S4a,b, and demonstrated in noise-free Fourier principal planes (Supplementary Fig. S5), the amplitude and phase embedding planes contain unique signatures for each Zernike mode. To illustrate the similarity of aberration-specific patterns across a variety of real-space samples, we apply 0.1 μm RMS of coma Z_3^{-1} to three different examples: a single bead (Supplementary Fig. S6a), a volume with five synthetic beads resembling training data (spanning $9.3 \times 9.3 \times 12.8$ μm^3, Supplementary Fig. S6b), and a volume of 100 synthetic beads resembling an AP2-labeled zebrafish embryo (corresponding to density beyond the parameters of the training data, Supplementary Fig. S6c). Despite drastic differences in real-space appearance, the Fourier embeddings (α_1, α_2, α_3, φ_1, φ_2, φ_3) remain consistent.

In contrast, real-space models trained on limited data (e.g., a few synthetic beads) did not generalize on dense puncta, where isolating individual features is difficult and aberrations are spatially variable. By moving to the Fourier domain, we leverage the OTF, which bounds the frequency space. Despite never seeing more than five beads during training, our model generalizes to volumes with 100+ resolvable puncta because each Zernike pattern persists in Fourier space (Appendix C.3) regardless of the real-space density.

Beyond generalization, Fourier embeddings reduces the inference cost by compressing the original isoplanatic patch from D×H×W to 6×64×64 voxels. A real-space model operating on 64^3 voxels requires more than ten times the computation. Further downsampling (to 6×32×32) accelerates inference but loses sensitivity to discriminate subtle changes in aberrations. Conversely, scaling up to 6×128×128 yields minimal gains for the first 15 Zernike modes, although may be necessary for higher-order modes.

During preprocessing, we also normalize phase embeddings to remove interference patterns introduced by sample structures (Supplementary Fig. S7d,e). Without phase information, the model cannot recover aberration signs, since amplitude alone lacks sign encoding (Supplementary Fig. S7f,g). While the principle amplitude (α_1) and phase (φ_1) planes capture lateral aberrations, additional planes (α_2, α_3 & φ_2, φ_3) are necessary for axial distortions (e.g., spherical aberrations Z_3^{-1}, Supplementary Fig. S7f). To capture such aberrations, we average a small set of planes along $\hat{k_z}$-axis, rather than using the orthogonal principal planes, to improve the model generalizability. A single model trained on the principal plane plus additional $\hat{k_z}$-axis planes then generalized across diverse lightsheet configurations with distinct axial support (Appendix C.2), enabling robust aberration prediction under varied imaging setups.

A.2 Synthetic training and validation data

We used the synthetic data generator described in Sec. 4.6 to create training and testing data for the first 15 Zernike modes, Z_0^0 through $Z_4^{\pm 4}$ (Supplementary Fig. S1). We show the λ RMS magnitude range for our training (blue) and testing (orange) distributions in Supplementary Fig. S21a. The panels show an example wavefront and a breakdown of each distribution: single (Supplementary Fig. S21b), bimodal (Supplementary Fig. S21c), Powerlaw—a heavy-tailed distribution with a few dominating modes (Supplementary Fig. S21d), and Dirichlet—uniform weighting across all modes (Supplementary Fig. S21e).

To train our models, we created two millions synthetic 3D volumes with aberrations ranging between 0.0 to 0.5 λ RMS. Each volume can have up to five beads with a uniform distribution of photons ranging between 1 and 200,000 integrated photons per bead.

We created a separate dataset with 100,000 volumes for testing. This smaller dataset extended the aberration magnitude up to 1.0 λ RMS, and the SNR range to 500,000 integrated photons. We also simulated samples with much higher object density (up to 150 objects in any given volume). We sample from this dataset to carry out individual test experiments described in Appendix C to stress test our models.

A.3 Multistage design

We used Fourier embedding ($\mathcal{E} \in \mathbb{R}^{\ell \times d \times d}$) as input to a vision transformer model with Ω stages (Supplementary Fig. S2a). For each stage, the ℓ Fourier planes are tiled into k patches (Supplementary Fig. S2b), applying the radial encoded Positional Embedding to each patch. These are passed through a sequence of n Transformer

(Supplementary Fig. S2c) layers, each consisting of h parallel multi-head attention layers (MHA), followed by a multi-layer perceptron block (MLP). At each stage end, a residual connection is added, and the patches are merged back into the shape matching the stage input (Merge patches). After all stages, the resulting patches are pooled (GlobalAvgPool) and connected with a dense layer to output the z Zernike coefficients.

To determine the optimal number of stages for our architecture, we designed several models by changing the number of transformer layers n used for each patch size p, while keeping the total number of transformer layers used in each model to 8, and choosing a patch size from $p_1 = 32$, $p_2 = 16$, or $p_3 = 8$.

Supplementary Fig. S3 shows our performance analysis of each model trained on the same dataset of 2 million synthetically generated samples. We created two models using a triple-stage design in Supplementary Fig. S3a, and three models using a dual-stage design in Supplementary Fig. S3b. We used 8 transformers for each model, but we vary the number of transformers used for each patch size.

The cost of running our triple-stage models is lower than our dual-stage models in terms of GFLOPs (Supplementary Fig. S3c), and both of our triple-stage models are smaller than the rest of the models we tested (Supplementary Fig. S3d). However, our dual-stage models converged to a better training loss, and were substantially faster in terms of training (Supplementary Fig. S3f), and inference (Supplementary Fig. S3h–k).

In general, using smaller patch sizes is more expensive, as the compute for self-attention layers scales quadratically *w.r.t.* the number of patches. Our analysis suggests that the smallest patch size $p_3 = 8$ is not necessary for our application, which is expected given that our starting input images are relatively small. Furthermore, the models that use more transformers for the biggest patch size ($p_1 = 32$) are faster, and perform better in terms of training loss. Given that most aberrations extend beyond (16×16) pixels in Fourier space, we chose a dual-stage layout with an equal number of transformers for each patch size ($p_1 = 32, p_2 = 16$) to balance between accuracy and training/inference time.

In all, we derived five variants—scaling the model size from 34 million up to 228 million parameters. Supplementary Table S2 shows the number of transformer layers n, number of heads h, embedding size ϵ, and MLP size x, highlighting the hyperparameters used to create each of our variants. Further evaluation for all variants of our model can be found in Appendix B—where they are also compared to other prominent architectures.

A.4 Training dataset size

In this section, we present a small case study to understand the scaling of our models in terms of model size, and training dataset size. For simplicity, we chose two models for this analysis: Tiny (T) and Small (S). We trained each model on a synthetic dataset that has up to eight million samples.

Supplementary Fig. S22 shows the cumulative distribution functions for our residuals after a single correction over 10,000 test samples with initial aberrations ranging from 0.2λ RMS up to 0.4λ RMS, simulated with 50,000 up to 200,000 integrated photons using a single bead. The colors indicate the number of training samples used for each model: 500K (blue), 1M (orange), 2M (green), 4M (red), 6M (purple), and 8M (brown).

The performance of our Tiny model starts to plateau after four million training samples—indicating it saturated its learning capacity given the limited number of trainable parameters that it can adjust during training (Supplementary Fig. S22a). On the other hand, our Small model continued to improve with more training data (Supplementary Fig. S22b). We show an additional analysis, scaling our model size up to 227 million trainable parameters in Appendix B.

A.5 Training hyperparameters

We trained all models on a two million sample dataset of synthetically aberrated beads, described in Sec. 4.6.2. Training uses Adam [42], augmented with decoupled weight decay regularization [43] with weight decay of 0.001, $\beta_1 = 0.9$, $\beta_2 = 0.99$, and a layerwise adaptive rate scaling [44] for faster training using large batch sizes.

We trained all models using a node with eight NVIDIA H100 GPUs for a total of 500 epochs with a two-phase learning rate scheduler—linearly increasing our learning rate from 0 to $1e^{-3}$ over 25 epochs (warmup phase), followed by a cosine decay [45] for the remaining epochs (decay phase). We used mean squared error (MSE) as our loss function with a batch size of 4096 unless otherwise noted. Supplementary Table S8 shows the breakdown of our training configuration.

We applied dropout [36, 46] with probability 0.1 after all Dense layers except the patch encoder layer [20]. We also used stochastic depth regularization with linearly increasing dropout rates and a max dropout rate of 0.1 [37].

We used Tensorflow [47] to implement our models. We derived five variants—scaling the model size from 34 million up to 228 million parameters—to compare our model design with the architectures described below. Supplementary Table S2 highlights the hyperparameters used to create each of our variants.

A.6 Measuring prediction confidence

To validate a single AOViFT prediction, we perform inferences on 361 digitally rotated copies of $\mathcal{E}$ through a range of 0—360 degrees and check if these predictions are self-consistent. For each Zernike mode $Z_n^{m \neq 0}$, there is a known rotational period, $\frac{2\pi}{m}$. Confidence in the prediction of each mode is established when the rotated inferences match the known Zernike modes' rotational periods, (Supplementary Fig. S23) within a desired tolerance. For rotationally invariant modes (e.g., spherical aberration), the variance of the Zernike amplitude must remain within a tolerance. When the model predictions exceed tolerance, the typical cause is there is not enough information in the data to make an accurate prediction for that mode. This will occur when the underlying sample structure does not have visible puncta (e.g., high spatial frequency content) or the acquisition has too much noise.

Supplementary Fig. S23a shows an example of our pipeline for measuring prediction confidence. The subpanels show the predicted amplitudes for each digital rotation, and a regression fit between the digitally rotated angle and the predicted twin angle to evaluate the variance in the predictions for 360 digital rotations.

Particularly, we show the predicted amplitudes for:

- Supplementary Fig. S23b: Oblique astigmatism ($Z_{n=2}^{m=-2}$, blue), vertical astigmatism ($Z_{n=2}^{m=2}$, orange), and the magnitude for both twin modes in black.
- Supplementary Fig. S23d: Vertical trefoil ($Z_{n=3}^{m=-3}$, blue), oblique trefoil ($Z_{n=3}^{m=3}$, orange), and the magnitude for both twin modes in black.
- Supplementary Fig. S23f: Vertical coma ($Z_{n=3}^{m=-1}$, blue), horizontal coma ($Z_{n=3}^{m=1}$, orange), and the magnitude for both twin modes in black.
- Supplementary Fig. S23h: Oblique quadrafoil ($Z_{n=4}^{m=-4}$, blue), vertical quadrafoil ($Z_{n=4}^{m=4}$, orange), and the magnitude for both twin modes in black.
- Supplementary Fig. S23j: Oblique secondary astigmatism ($Z_{n=4}^{m=-2}$, blue), vertical secondary astigmatism ($Z_{n=4}^{m=2}$, orange), and the magnitude for both twin modes in black.
- Supplementary Fig. S23l: Predicted amplitudes of primary spherical for each digital rotation ($Z_{n=4}^{m=0}$, blue).

Supplementary Fig. S23[c, e, g, i, and k] show the result of our regression fits. If the mean residual error (MSE) of these predicted twin angles exceeds 700 and the magnitude of the mode is above 0.05 μ *RMS*, then we flag that prediction as notconfident (e.g., , Supplementary Fig. S23c, highlighted in red). We highlight confident predictions in green (e.g., Supplementary Fig. S23e, and Supplementary Fig. S23k). If our MSE for the digital rotations is high but the magnitude of the mode is below 0.05 μ *RMS*, then we mark these modes in blue as confident zero predictions (e.g., Supplementary Fig. S23g and Supplementary Fig. S23i). We show the final prediction for each Zernike mode in Supplementary Fig. S23m, and the corresponding wavefront in Supplementary Fig. S23n.

B ARCHITECTURE BENCHMARK

B.1 Baseline models

We compared our model architecture with two widely used backbones, namely transformer-based ViT [20], and convolution-based ConvNeXt [41] as shown in Fig S8.

ViT [20] originally was used for 2D RGB images and later adopted to 2D RGB videos [22]. To compare with our model, we extended the ViT architecture to our use case (3 spatial dimensions) by replacing each transformer layer with a new building block to process 3D volumes. Following the configurations proposed in [48], we created the variants:

- ViT-Small (layers=12, heads=6, EMB=384, MLP=1536),
- ViT-Base (layers=12, heads=12, EMB=768, MLP=3072),
- ViT-Large (layers=24, heads=16, EMB=1024, MLP=4096),

trying both 16 and 32 as the fixed patch size for all transformer layers in the variant. We set the dropout rate to 0.1 for all variants [36, 37].

ConvNeXt [41, 49] was also designed for 2D RGB images and videos. To adopt the model backbone to 3D spatial data, we replaced each 2D convolutional layer with a 3D convolution, without any other modifications to the rest of the architecture. We used a kernel size of (1, 7, 7), a downscale ratio of (1, 2, 2) for each stage, and stochastic depth rate of 0.1 [37]. As described in [41], we create four variants of the model:

- ConvNeXt-Tiny (C=[96, 192, 384, 768], B=[3, 3, 9, 3]),
- ConvNeXt-Small (C=[96, 192, 384, 768], B=[3, 3, 27, 3]),
- ConvNeXt-Base (C=[128, 256, 512, 1024], B=[3, 3, 27, 3]),
- ConvNeXt-Large (C=[192, 384, 768, 1536], B=[3, 3, 27, 3]),

where C is the number of channels, and B is the number of inverted bottleneck blocks [50] for each stage in the model.

B.2 Cost analysis

To evaluate the performance of these models, we used a subsample of our testing dataset with single beads only (Sec. 4.6.3). Particularly, we use 10,000 samples with aberrations ranging between 0.1λ to 0.2λ RMS—simulated with 50,000 to 200,000 photons. For each sample, we made three iterative corrections, measuring the RMS residuals of the aberration after each iteration. We show the median RMS residuals for all samples after two corrections in Supplementary Fig. S8a. All models tested here, except for ConvNeXt-T, were able to reduce the residuals down to the diffraction-limit (i.e., below $\approx 0.075\lambda$ RMS or $\lambda/4$ peak-to-valley [10, 11]) using two corrections only. Supplementary Fig. S9 shows the results for all three corrections.

While equivalent accuracy is found between model variants, it is critical to choose the model with the optimal set of trade-offs, rather than using the model with marginally better loss (Supplementary Fig. S8B). Since all models are capable of reducing the aberration down to diffraction-limit with a reasonable number of iterations, we compare these models across three salient factors: floating point operations per second (FLOPs); model size (number of parameters and memory footprint); speed (training time, throughput, and latency).

While our models' FLOPs for training (Supplementary Fig. S8c) and inference (Supplementary Fig. S8i) are slightly higher than ViT/32 because of the overhead of our multistage design, FLOPs

of our models are still better than ViT/16 and ConvNeXt. A model with lower FLOPs does not necessarily mean a better model overall, because the measurement of FLOPs does not factor in relevant details encoded into the architecture like parallelizable operations and the cost of any individual operation for a given hardware [51]. Since all models are trained on the same hardware, we can look at training time to measure the efficiency of these architectures. Our models' training hours (Supplementary Fig. S8d) are better because our design enables us to use a large batch size for training (Supplementary Fig. S8e), while maintaining a small memory footprint (Supplementary Fig. S8f), and a lower number of trainable parameters (Supplementary Fig. S8j).

ViT uses a fixed patch size for all of its transformer layers. Thus, using a smaller patch size (e.g., ViT/16) will increase the sequence length (i.e., number of patches) being processed by each layer, scaling the computational cost for self-attention quadratically for all transformer layers in the model. Our multistage architecture allows our models to converge faster by learning Fourier patterns from several scales, and also reduces the cost of our models by leveraging cheaper transformer layers with bigger patch sizes.

Unlike ViT, our embedding size ϵ_i changes based on the number of voxels per patch in each stage i. Our initial stage has a patch size $p_1 = 32$, resulting in an embedding size $\epsilon_1 = 1024$. While our second stage uses a smaller patch size $p_2 = 16$, our embedding size $\epsilon_2 = 256$ is significantly smaller, forcing the model to compartmentalize and compress the learnable parameters of deeper layers. Therefore, our transformer layers with smaller patch sizes have a smaller embedding size, instead of keeping the embedding size fixed throughout the model. This design scales particularly well as we increase the model size up by adding transformer layers (Supplementary Fig. S8k), and increasing the number of transformer heads (Supplementary Fig. S8L) for each layer.

In terms of inference speed, we see an advantage of using our models over the other models tested here. Our model variants perform exceptionally well *w.r.t.* throughput—maximum number of predictions per second using a batch size of 1024 (Supplementary Fig. S8g), and latency—average inference time (milliseconds) per image using a single NVIDIA A100 GPU (Supplementary Fig. S8h). Table. S3 shows a breakdown of these cost indicators for all models tested here. Based on this analysis, we select our small model for use on experimental data.

C IN-SILICO EVALUATIONS

C.1 Sensitivity to SNR

To test our model's sensitivity to SNR, we evaluated our Small model on 10,000 test samples using a single bead with a mixed distribution of aberrations of magnitudes up to 1 λ RMS. Supplementary Fig. S10a shows the initial aberrations in the test dataset as a function of integrated photons. Supplementary Fig. S10b–f shows five rounds of corrections.

We also show first round corrections for specific single modes of aberrations under low SNR conditions (i.e., $\leq 100{,}000$ integrated photons) in Supplementary Fig. S24—S25.

Supplementary Fig. S24a shows the residual λ RMS after a single correction for vertical astigmatism $Z_{n=2}^{m=2}$, along with XY MIPs of the residual aberration for various aberration amplitudes and SNR (Supplementary Fig. S24b). Max counts is highlighted above each PSF.

For single mode aberrations of $\leq 0.2\,\lambda$ RMS amplitude, the model can reach diffraction-limited performance for as low as 20,000 photons. However, it is exceptionally difficult to get good corrections for large aberrations at this SNR. We see similar trends for vertical trefoil $Z_{n=3}^{m=-3}$ (Supplementary Fig. S24c), vertical coma $Z_{n=3}^{m=-1}$ (Supplementary Fig. S24e), oblique quadrafoil $Z_{n=4}^{m=-4}$ (Supplementary Fig. S25a), oblique secondary astigmatism $Z_{n=4}^{m=-2}$ (Supplementary Fig. S25c), and primary spherical $Z_{n=4}^{m=0}$ (Supplementary Fig. S25e).

C.2 Generalization to other light sheets

To evaluate our model's generalizability to other light-sheets, we created four synthetic datasets with 10,000 samples each having aberrations up to 1 λ RMS—using a single bead with up to 500,000 integrated photons.

Supplementary Fig. S20a–c shows the residuals for three rounds of corrections using the same multi-Bessel LLS as used for generating the training data. The next row (Supplementary Fig. S20d–f) shows the results for a LLS of the same type but at higher NA. It achieves similar performance for the same training set, despite the higher NA.

However, the model's performance degrades when applied to Sinc (Supplementary Fig. S20j–l), and Gaussian (Supplementary Fig. S20m–o) light sheets, as these have notably different cross-sectional profiles. Supplementary Fig. S20I–V show the excitation profile used to create each LLS *w.r.t.* the length of each LLS in microns. Supplementary Table S6 shows the LLS specifications we used for this test.

C.3 Sensitivity to the density of objects

Our model was trained with up to five simulated beads in any given FOV. However, most experimental data will have dozens if not hundreds of objects in a single volume. Therefore, we evaluated our model on a dataset of 10,000 samples simulated with up to 150 beads to understand its sensitivity to the density of objects ($\bar{\delta}$nm).

Supplementary Fig. S13 shows the residual λ RMS for five rounds of corrections as a function of the average distance to the nearest bead in that given FOV. The results suggest the model generalizes to a much larger number of objects than it was trained on. The

heatmaps (Supplementary Fig. S13b–f) show continuous improvement over 5 iterations provided there is not much overlap among the objects ($\bar{\delta} \geq 200$nm).

C.4 Sensitivity to object size

As mentioned in Sec. 4.6, we simulated individual objects (beads) as Gaussian kernels. We uniformly chose the kernel's full width at half max (FWHM) for each object between 100nm and 400nm to create our training dataset.

Here, we show a test of the extent to which our model can reliably correct individual aberration modes of 0.3 λ RMS amplitude *w.r.t.* object size.

Supplementary Fig. S26a–b shows the residual λ RMS after a single correction using our Small model (S) for vertical astigmatism $Z_{n=2}^{m=2}$ simulated with 50,000 and 100,000 integrated photons, respectively.

We also show representative XY MIPs of these aberrations using bead kernels of FWHM 100nm, 200nm, 300nm, and 400nm, respectively (Supplementary Fig. S26I–IV). We find that as the objects get larger, the unique signature of these aberrations in real space and Fourier space is degraded. Better SNR helps improves correction by detecting smaller fringes in the Fourier embedded input images, but the ability to correct for non diffraction-limited objects still depends on the nature of the aberrations. As a rule of thumb, our model can detect aberrations as long as they are reasonably apparent in the real space images. Supplementary Fig. S26c–f show results for vertical trefoil $Z_{n=3}^{m=-3}$, and vertical coma $Z_{n=3}^{m=-1}$, respectively.

D GENOME EDITING OF AP2S1 FOR ZEBRAFISH

Genome editing of *ap2s1*, *ap2s1:ap2s1-mNeonGreen*^{bk800} in zebrafish was performed using a combination of previously described techniques [52–54]. First, a 2.4 kb DNA fragment that spanned the stop codon of *ap2s1* was cloned from AB wild type genomic DNA (genomic DNA from the AB population at UC Berkeley). The 2.4 kb PCR fragment was designed to include 1.2 kb of the end of *ap2s1* and 1.2 kb downstream of the stop codon that were the homology arms for homology dependent repair. The fragment was sequenced to ensure that the selected guide RNA target was present and lacked any SNPs. The target sequence of the guide RNA used to cut *ap2s1* was ACTGATTGACAGTTTACTCC (identified using the CRISPscan [55] software, guide RNA synthesized by IDT). The donor did not require any mutations to prevent cutting because the guide target spanned the stop codon of *ap2s1*. The mNeonGreen used was designed and synthesized to minimize predicted RNA splicing donor and acceptor sequences without changing the protein [56]. Isothermal assembly was used to clone a linker and mNeonGreen green sequence before the stop codon of *ap2s1* [57]. The backbone we cloned the genomic fragment into and engineered the donor within contains a set of flanking sequences that we used for both isothermal assembly and PCR of the genome editing donor. These sequences come from the inDrops single-cell transcriptomics library preparation protocol and worked well for efficient PCR (GCATACGAGATCTCTTTCCCTACACG, CACGGTCTCGGCATTCCTGCTGAAC) [58]. For making an HDR donor for genome editing, oligos with biotin added to the 5' ends were used to prevent oligomerization upon injection [54]. For embryo injections, 2 nl was injected into the 1-4 cell embryo of our injection recipe: 5μM guide RNA (purchased from IDT), 50 ng/μl clean cap Cas9 mRNA (from Trilink), 100 mM KCl, 0.1% phenol red, 0.1 mM EDTA, and 1 mM Tris, pH 7.5. Approximately 3000 embryos were injected by a group of 3-5 lab members. Injected embryos were screened and selected for health, and successful injections as indicated by broad fluorescence signal were raised to adulthood. After 2-3 months, zebrafish were screened for potential founders through germ-line transmission. Embryos with positive signal were established as a new allele once we confirmed by PCR that mNeonGreen had been inserted cleanly at the targeted site in *ap2s1*. Founders were out-crossed with Casper (mitfa$^{w2/w2}$; mpv17$^{a9/a9}$) [59] mutants, and their offspring were crossed with Roy Nacre mutants again to establish the heterozygous *ap2s1-mNeonGreen* and homozygous Casper alleles. These fish were in-crossed and screened for homozygous *ap2s1-mNeonGreen*$^{bk800/bk800}$ embryos for imaging.

DNA Sequences used for zebrafish genome editing and labeling mitochondria

- GCATACGAGATCTCTTTCCCTACACG —forward biotinylated primer for generating HDR template by PCR
- tgctaaagttgactg... —left homology arm
- gggcGGatccggtggatccggtggatct —GGS linker between Ap2s and mNG polypeptides
- gtTagCaagggcg... —mNeonGreen through to stop codon
- actgtcaatcagtc... —right homology arm

- GTTCAGCAGGAATGCCGAGACCGTG—reverse biotinylated primer for generating HDR template by PCR

GCATACGAGATCTCTTTCCCTACACG tgctaaagttgactgttgagttt
tacagtgtagttagtgttttacactatcccaaaataagtctgatcattgttttactaaggcatgt
gaaatatttattatcgactaaaatattgttattgttgtcaatatgtgagctcatgtttagtatgcc
attcattttgtcaaaaataaatgaataaaaaaaaaacatgtttagagagtcagcaagaatatac
tatgaaacttattagaaggcagttaaaaagatcaattcaatatataaaagcaaaaacatatac
ggttgaagtcagaattattagcccccttttgaatttttttttctttttttaaatatttcccaaatgatg
tttaacagagcaaggaaactttcacaatatgtctgattatattttttcttctggaaaaggtctttc
ttgttttatttcggctagaataaaagccatttttaatttttaaacaccattttaaggacaaaattat
tagccccttaaaggtaatttttttttttttacgatagtctacagaaccattgttatacaataacttg
gctaattaccctaacctgcctagttaacctaattaacatacacgcatacataccttttaacacg
catacaaacacacacaatttttattttcacaaaataatgtttttgaaattcccagagatgggttg
cggccggaagggcatctggtgtgtaaaaatgtgctggataagttggcggttcattccgctgt
ggcgaccccagataaataaagggactaagccgacaagaaaattaatgaatgaacgaatga
atgtttttgaagtttaagtttgtgtagaactgcgttatagtcaagaaatatcccaaaaattgagt
catgttgacaaacatacattgattaagttaacttaattgtttttgcaaattaaagtggattgga
actaaagcaattaatttttctctcaaaaaacttaagaattgatttagctcaactaacaaaaata
tttttgagtgcaaacacatgtaaaaaatgcacgaagcagcaagtttttagttgaaaacagcg
gccctatatgtgaactgctattaagaagtattgtggatgtgtttcaggtgtatacggtggtgg
acgagatgtttctagcaggagagatcagagaaaccagccagacgaaggtgctcaagcag
ctcctcatgctgcagtccctgga gggcGGatccggtggatccggtggatct gtTagCa
agggcgaggagGACAAtATGGCCTCtCTGCCCGCAACACACGAG
CTGCATATTTTCGGAAGCATCAAcGGcGTGGATTTCGAtATG
GTtGGgCAaGGaACTGGAAACCCAAATGAcGGaTACGAGGAA
CTGAATCTGAAGTCAACCAAAGGCGACCTCCAaTTCtcaCCT
TGGATTCTcGTtCCCCAtATTGGCTATGGaTTTCATCAaTATC
TGCCaTAtCCTGAtGGAATGTCACCATTTCAaGCcGCTATGGT
GGATGGATCTGGCTACCAaGTCCACcgcACCATGCAaTTTGA
GGACGGcGCCtccCTGACTGTGAACTACCGCTATACCTACGA
GGGATCtCATATCAAGGGCGAAGCACAaGTtAAAGGaACAG
GATTCCCAGCTGAcGGCCCCGTCATGACAAACTCTCTGACC
GCCGCCGACTGGAGCCGGTCCAAGAAAACTTACCCTAACG
ATAAGACCATCATCTCTACCTTCAAaTGGAGTTATACCACc
GGCAACGGaAAGCGcTACAGAAGCACAGCCCGAACTACCT
ATACTTTTGCTAAGCCtATGGCTGCAAACTATCTGAAAAAT
CAGCCTATGTAtGTcTTCcGAAAaACcGAattgAAGCACTCCAA
AACAGAACTGAATTTCAAGGAgTGGCAGAAGGCTTTTACC
GATGTtATGGGcatggacgagctgtacaaAtaaactgtcaatcagtcaaccaacca
atcaaacaaccaccattgtgtcaggctccgcccaccccgctgcagccaagccatatgatttgt
gtgtatgagtgttgtcatgctgaaaatcatcctctaacatttcacctttttctttacactcgcatc
cacctcacaggatgttgccgttctgtcctgcaacttcatgaattttcgaggcagataagaattc
gtaaaacttcctcggttatgtgacctgattatatcttggcatagccagaattgaattcttcagcg
attttatagcacactagacttcagtgtttccgactggaagcctctccaagcctgttcattaacac
taacatagataatagcgtcatgtgggccaaaaaactgccatggcacgaaagtgacagtcat
aattgcaagattccggggctgcactgcaatatcgcaatgagaaaaacttgatttgatgtccat
ttaaggaaattcccatatatgtactattaaaaaaagaatcaaatgtttaatatgctgcactgca
aataataaatttgttactgctttgtcttgttttctggtacaagtatccatattgttaaaactccaaa
tacgtaaaggtgatcttcctgttggtgactatatctttttttatacaattaagatgttcataatgg
tgtcacaaaaagggtttcgcaacaactgttggcacttttgagagtaaaaaaaaaacatacag
gcaaaaccaaattaactgaccgtttcagtatggagatgtcattaatccatgaacagttcctgct
tgtcttaactatttctgaattgtaagaggcgattcggttgtaactaatgttaacatggctgctaa
aaaaagtgtcaatatggacctttctggattctaataagatttggaaatgaagatataaaacag
caaatacataaataccattgttattatttacatccctcaaaatggtctacccactactgattcgg
tgaggtcctaagaagtgaaattaaaatttatgtcaaacaaaatacattattttagatccacagc
tttggccaattgtattctttttgttttgcctattattattattttttttttttcactctaaaaagtaata
atcgggttaatcagcctaatggtgaatttaaagaaaaatgaaggagaaaacaaaatatttcc
ttgccatactcttac GTTCAGCAGGAATGCCGAGACCGTG

COX8a-mChilada (mitochondria)

- ATGTCTGGACT…—cDNA of N-terminal 34 amino acids of *Cox8a*
- GACAATATGG…—mChilada
- ggcGGatccggtgg—linker

ATGTCTGGACTTCTGAGGGGACTAGCTCGCGTCCGCGCCG
CTCCGGTTCTGCGGGGATCCACGATCACCCAGCGAGCCAA
CCTCGTTACGCGACCCGCGAAG ggcGGatccggtggatccggtgga
AGCgtTagcaagggcgaggag GACAATATGGCTATTATCAAAGA
ATATATGCGTTTTAAaGTtCATATGGAGGGCAGCGTCAATG
GACATGAATTTGAAATTGAGGGcGAGGGAGAGGGCAGGCC
ATTCGAAGGGACTCAGACAGCGAAGTTGAAAGTTACCAAG
GGGGGACCATTGCCATTTGCATGGCACATCCTCCCGCCCCA
aTTTCAATATGGTTCTAAGGCATATGTTAAGCATCCTGCTG
ATATACCCGATTACTTCAAaCTgTCaTTTCCtGAgGGTTTTAC
CTGGGAACGCGAAATGAATTTTGAAGACGGCGGCGTtGTA
ACAGTCACACAGGACTCCAGTCTGCAaGACGGCGAGTTTAT
CTACAAAGTtAAACTTCGTGGAACCAACTTTCCCTCCGACG
GGCCTGTCATGCAAAAGAAGACAATGGGGAACACCGCTTC
AACcGAGCGtATGTACCCGGAAGATGGAGCTCTTAAgGGcG
AAACGAAGTGGCGTCTTAAGTTGAAAGATGGTGGTCACTA
CGAGGCTGAAGTGAAGACCACGTATAAGGCGAAAAAGCC
CGTACAGCTGCCTGGAGCGTACAATGTGGATCGTAAATTG
AAGATAACCTACCATAATGAGGATTACACCATCGTGGAGC
AGTATGAACGAGCCGAGGCTCGGCACTCAACGGGTggcatgg
acgagctgtacaagtaA

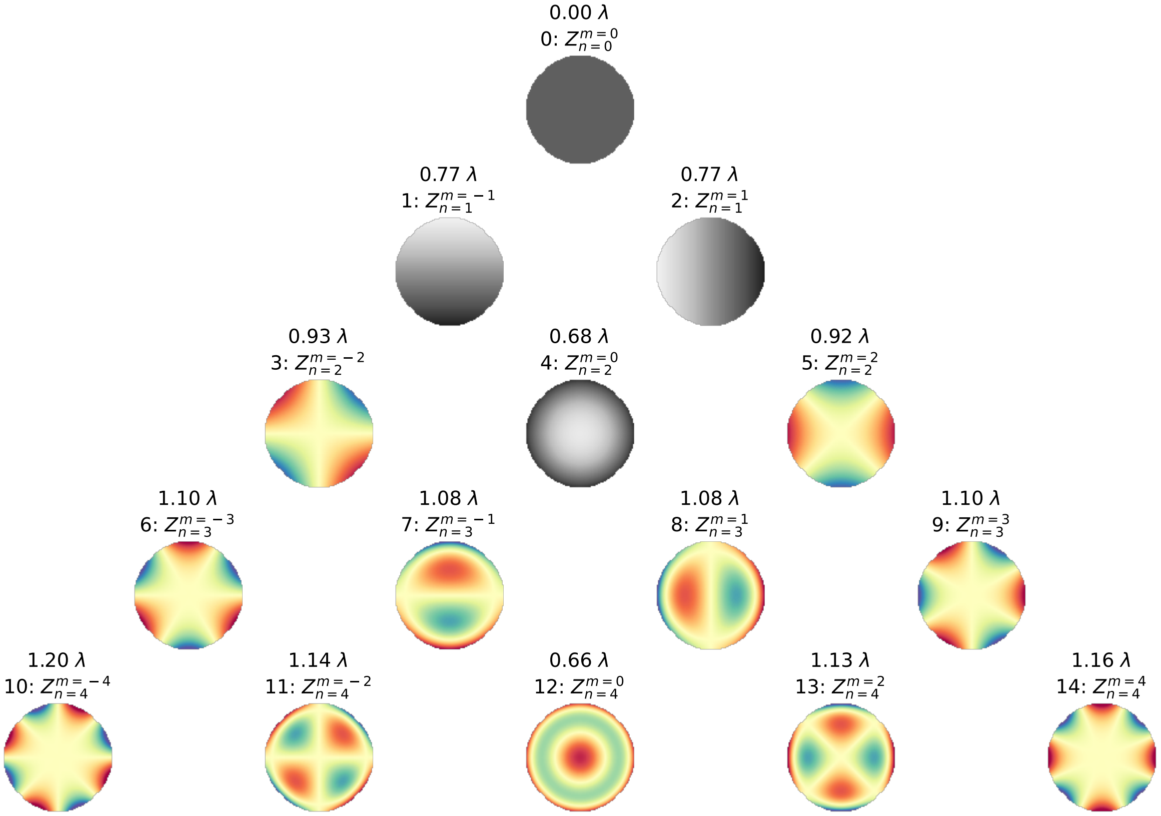

Figure S1: Zernike modes (OSA/ANSI standard indexing). Wavefronts for the first 15 Zernike modes (Z_0^0 through $Z_4^{\pm 4}$) with 0.1 μm RMS applied to each mode. Undetectable modes (bias, tip, tilt, and defocus) are greyed out, and the measured peak-to-valley for each mode is reported in waves for a wavelength $\lambda = 510nm$.

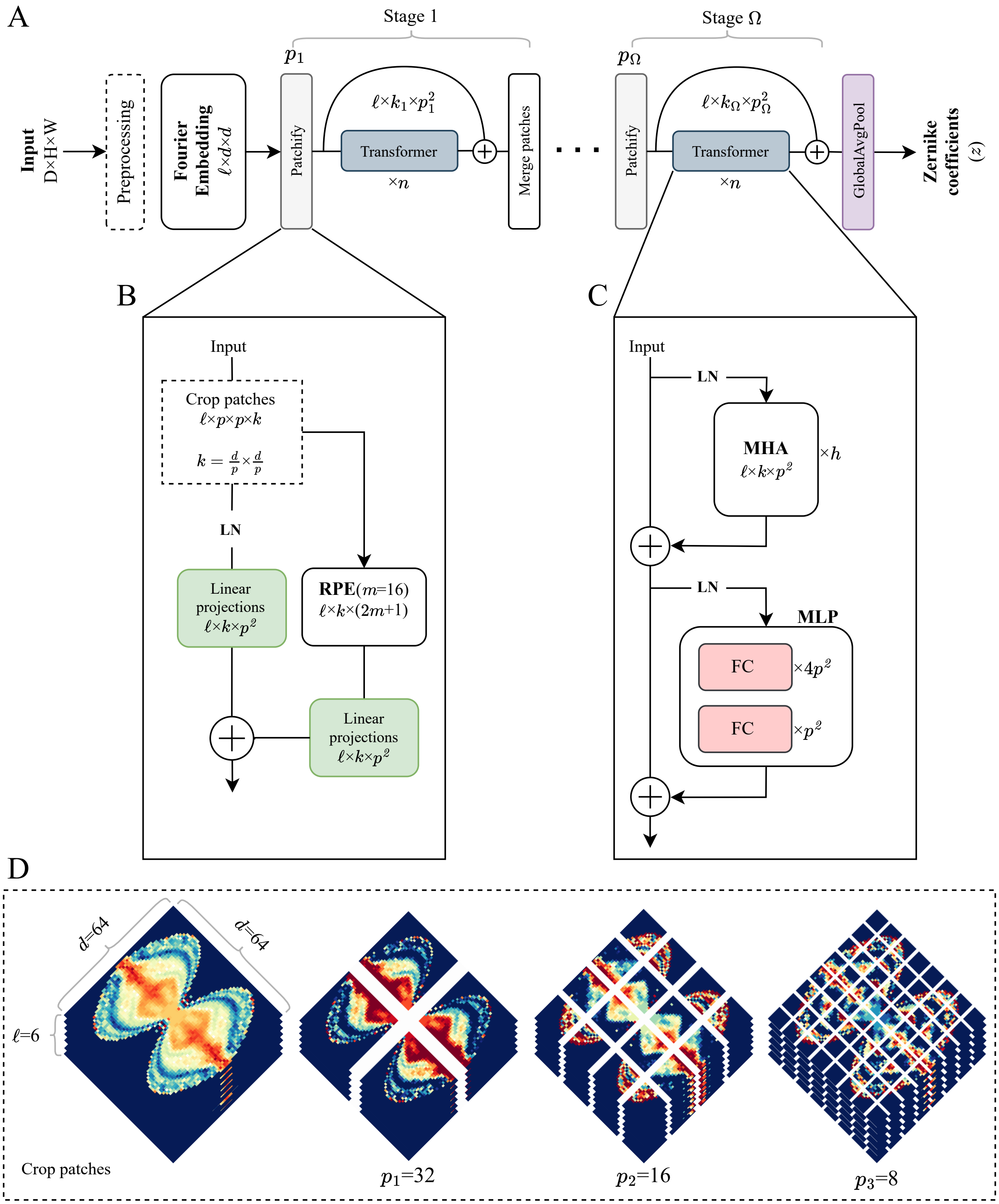

Figure S2: Multistage architecture. **A**. A schematic of the general form of our model with Ω stages. **B**. A breakdown of the patchify layer. **C**. A breakdown of the transformer layer. **D**. An example output of the crop patches block using a patch size of 32, 16, and 8, respectively.

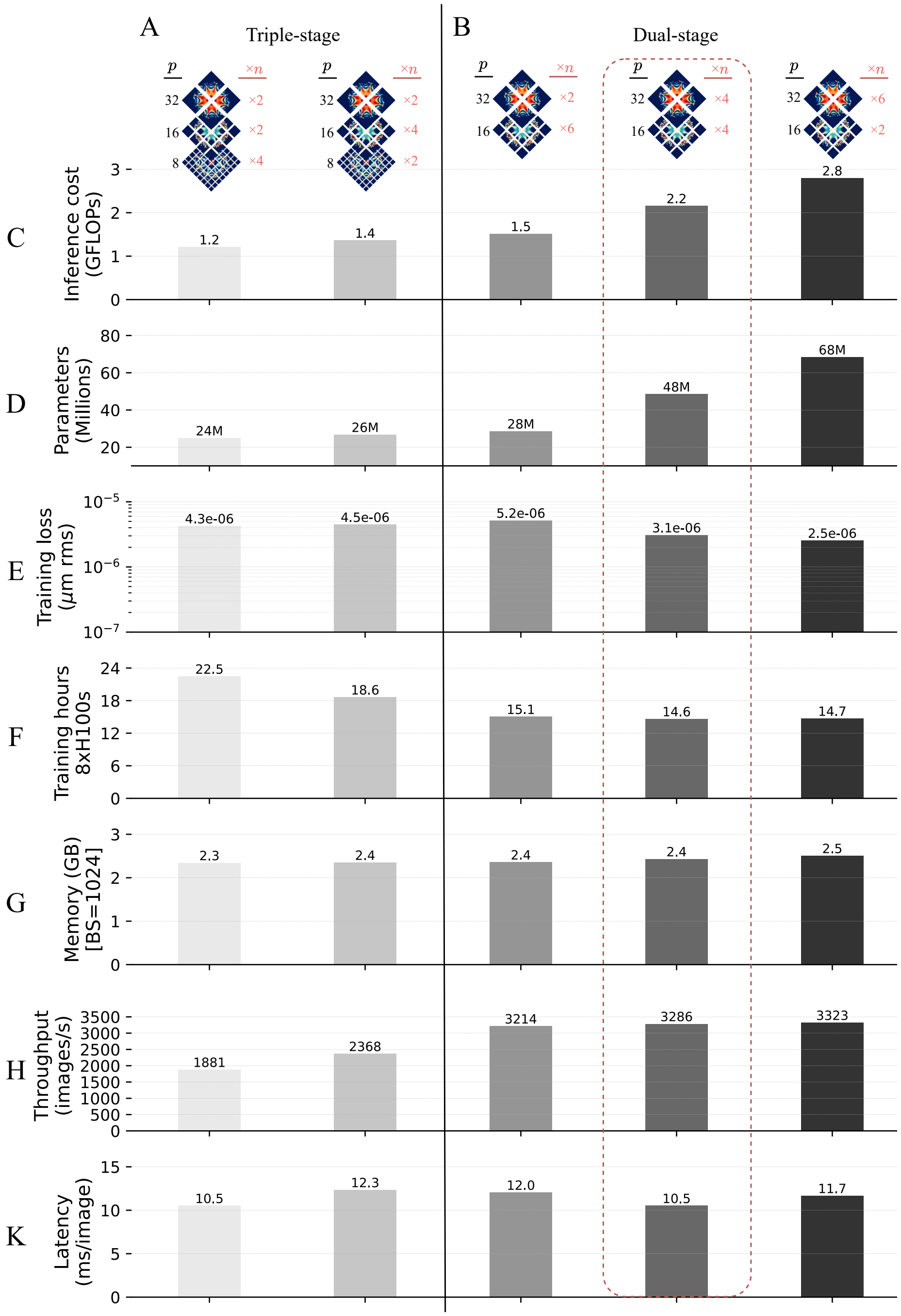

Figure S3: Performance analysis of our multistage design using a total of $n = 8$ transformer layers, and several patch sizes $p_1 = 32$, $p_2 = 16$, and $p_3 = 8$. **A**. Triple-stage design with a patch size of 32, 16, and 8 respectively. **B**. Dual-stage design with a patch size of 32 and 16. **C**. Inference cost per image measured in GFLOPs (10^9 FLOPs). **D**. Total number of trainable parameters. **E**. Training loss using a dataset of 2M synthetically generated samples. **F**. Training time using a single node with eight H100 GPUs. **G**. Memory footprint of each model with a batch of 1024 images using 16-bit floating point precision. **H**. Throughput (average number of predictions/images per second) using a batch size of 1024 on a single A100 GPU. **K**. Latency (average inference time per image) measured in milliseconds for a batch of 1024 examples using a single A100 GPU.

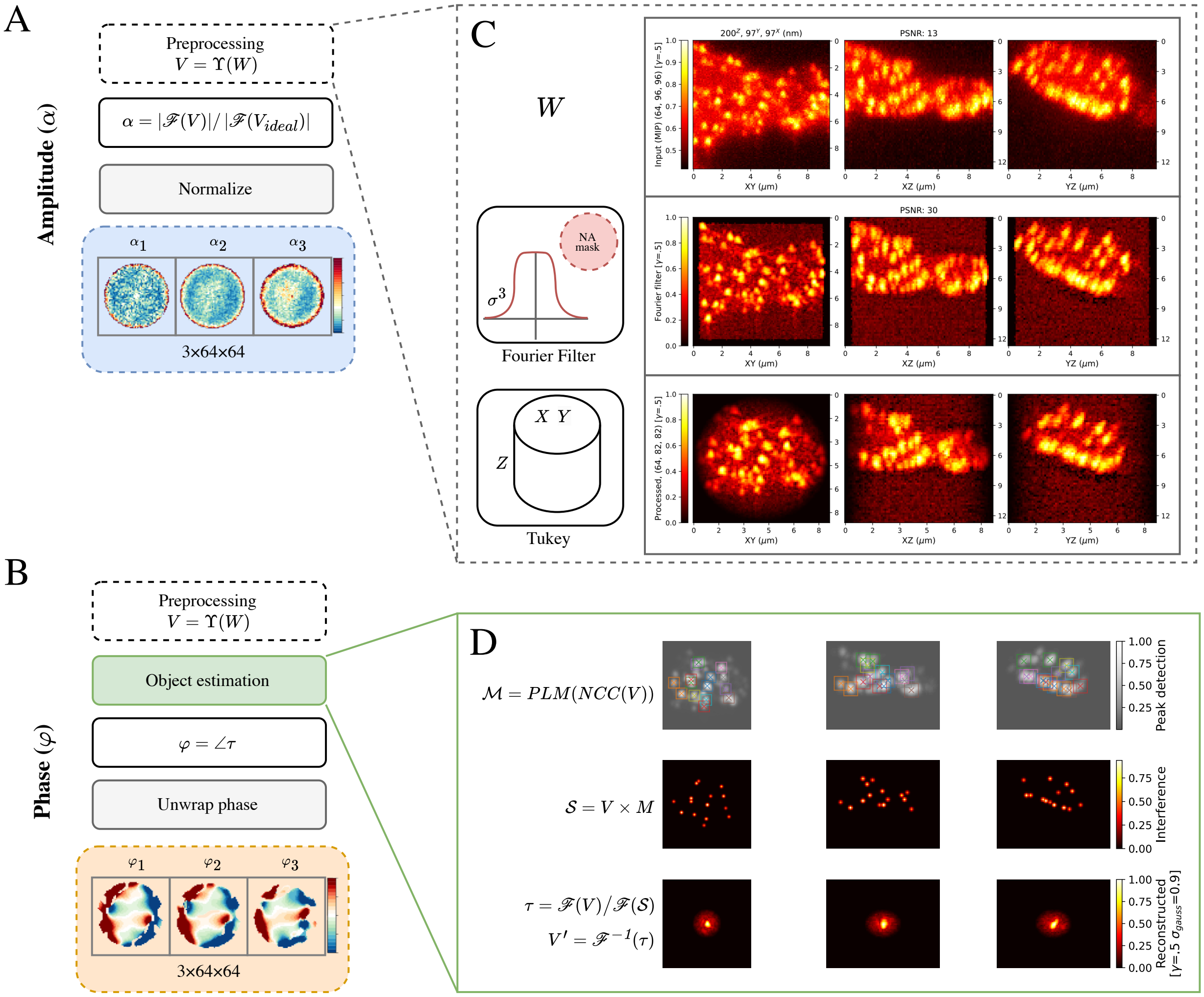

Figure S4: Conversion of raw image volumes to Fourier embeddings. The raw volume is first frequency filtered (Fourier Filter) and windowed (Tukey window, $\hat{x}\hat{y}$ only). The 3D FFT of the result is input into the Amplitude (α) and Phase (φ) Fourier Embedding calculations. **A.** A breakdown of the steps needed to create the amplitude embedding (α). **B.** A breakdown of the steps needed to create the phase embedding (φ). **C.** MIPs from a zebrafish embryo covering $9.3 \times 9.3 \times 12.8$ μm^3 FOV to illustrate the preprocessing modules we use to reduce noise and suppress edge artifacts. **D.** MIPs of the same FOV showing the intermediate steps to remove the interference patters from the given volume.

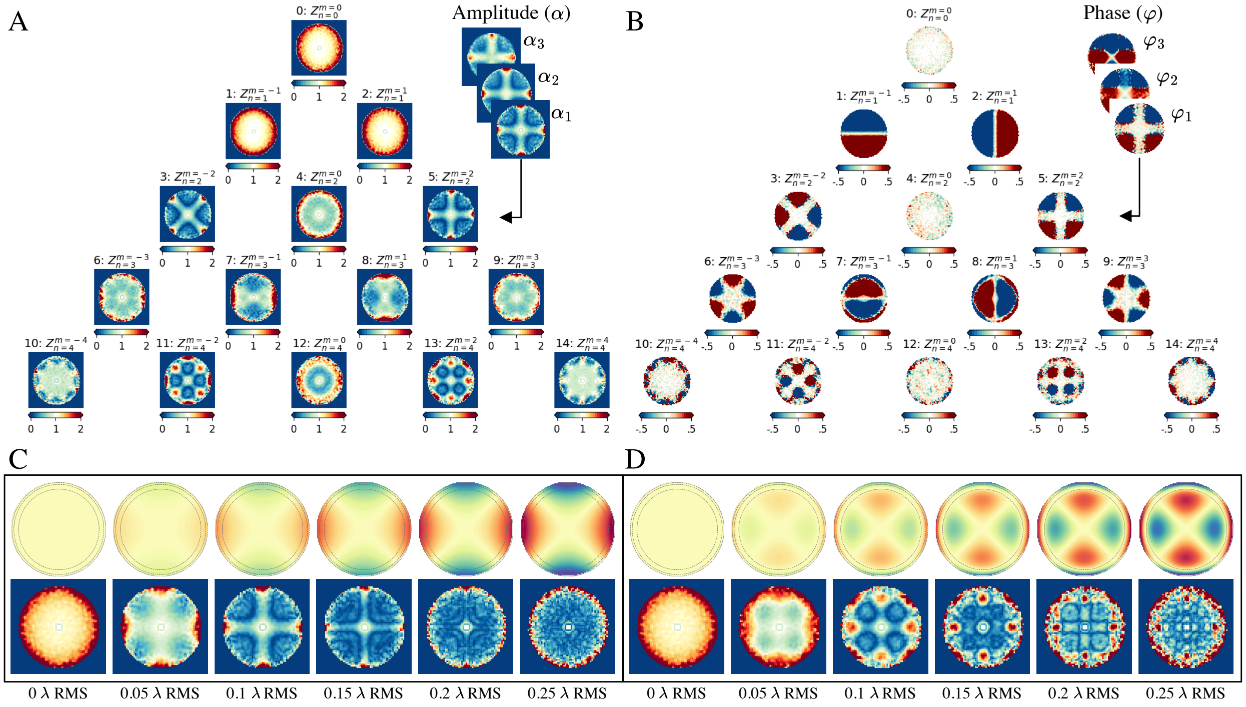

Figure S5: Aberration fingerprints. The principal Fourier plane of **(A)** amplitude (α_1) and **(B)** phase (φ_1) embeddings for the first 15 Zernike modes (Z_0^0 through $Z_4^{\pm 4}$) each at 0.1λ RMS, shown for an ideal noise-free volume containing a single point emitter. The wavefronts and corresponding amplitude embeddings (α_1) for **(C)** Z_2^2 and **(D)** Z_4^2 as aberration magnitudes increase from 0 to 0.25λ RMS.

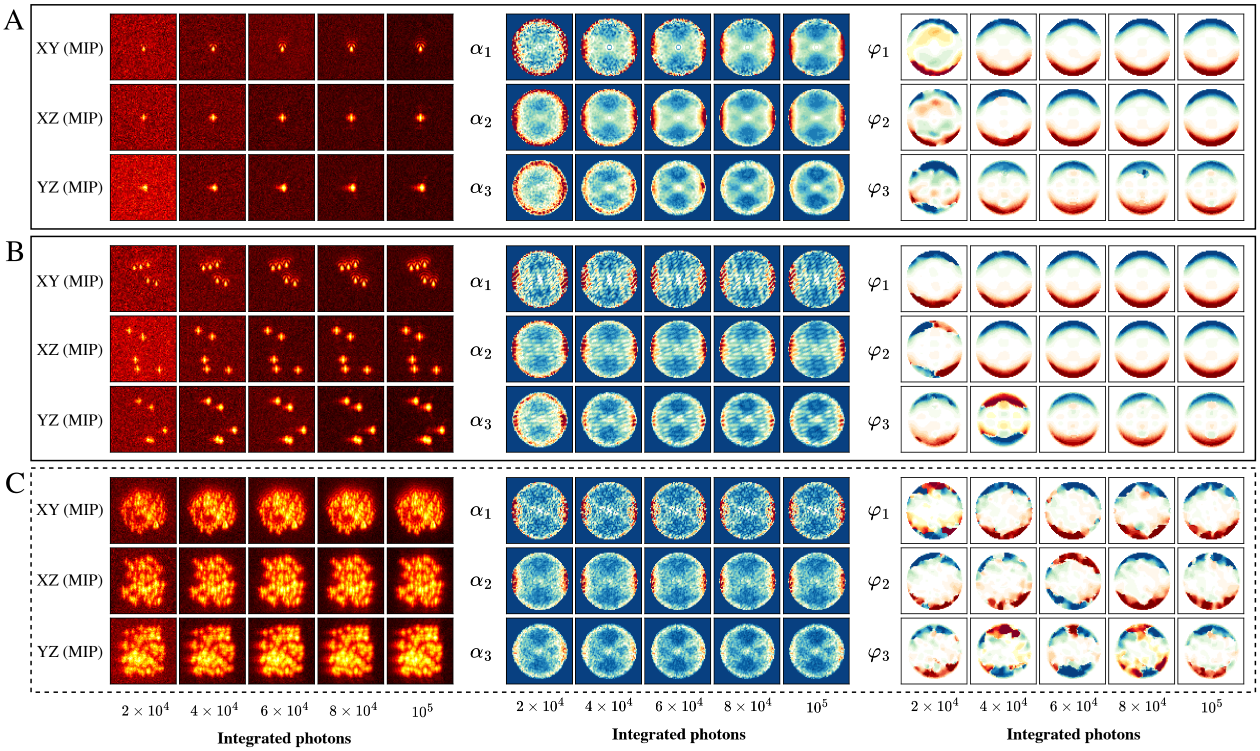

Figure S6: Generalized representations via Fourier embedding. Real-space (left) versus Fourier amplitude (middle) and phase (right) embeddings (α_1, α_2, α_3, φ_1, φ_2, φ_3) for a $9.3 \times 9.3 \times 12.8$ μm^3 volume. An aberration of 0.1λ RMS in mode Z_3^{-1} is applied to volumes containing **(A)** a single bead, **(B)** five beads, and **(C)** one hundred beads.

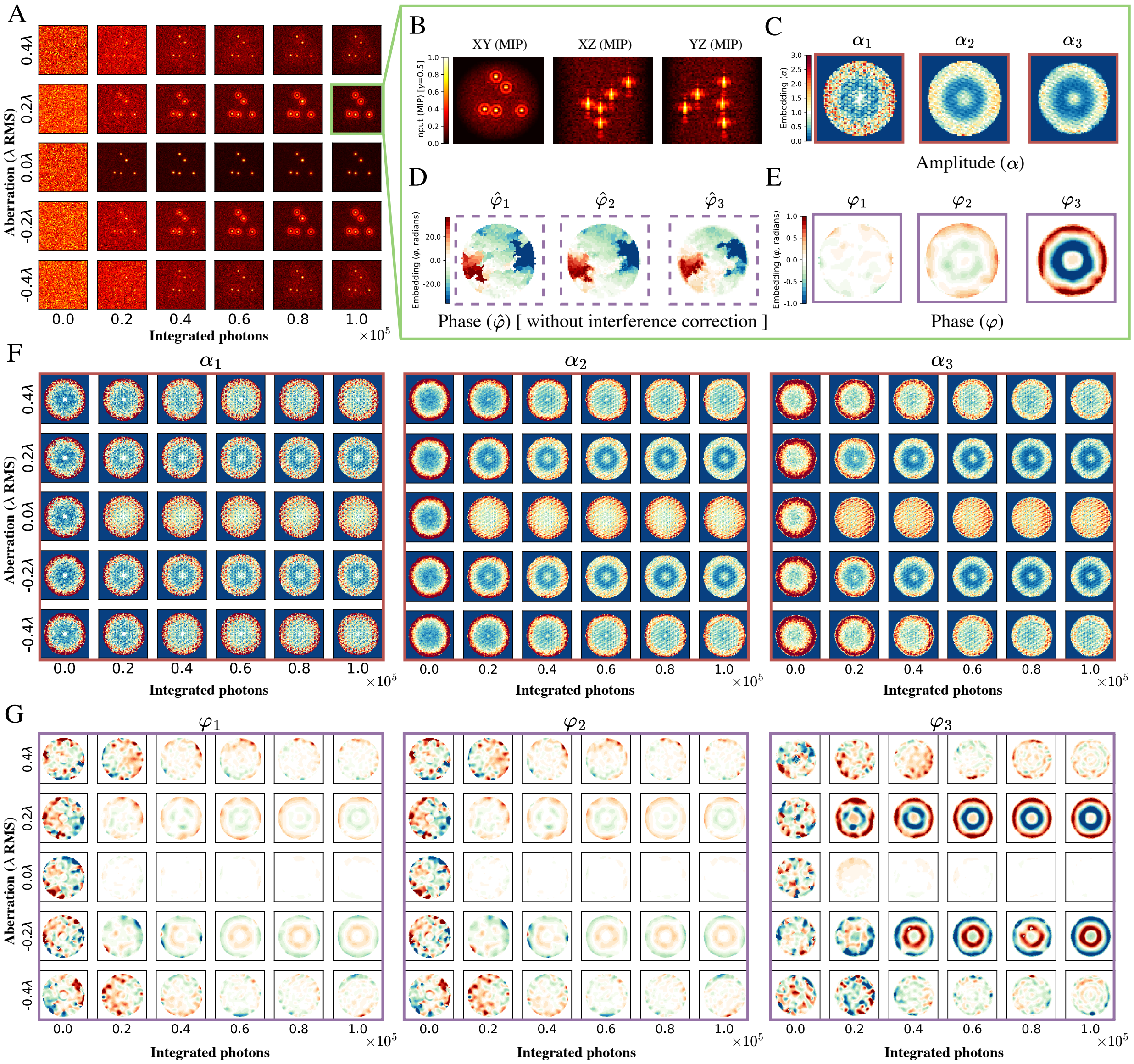

Figure S7: Fourier embedding composition. **A.** XY MIPs of five synthetic beads, each with an initial aberration ranging from -0.2λ RMS to 0.2λ RMS in mode $[Z_{n=4}^{m=0}]$ at different SNR levels. **B.** XY, XZ, and YZ MIPs of a single FOV simulated with 10^5 photons. **C.** Amplitude embedding (α_1, α_2, α_3) for the FOV in **B**. **D.** Phase embedding (φ_1, φ_2, φ_3) for the same FOV in **B**, shown without interference correction. **E.** Phase embedding after after removing bead-induced interference patterns. **F.** Amplitude embeddings for all examples shown in **A**. **G.** Phase embeddings for all examples shown in **A**.

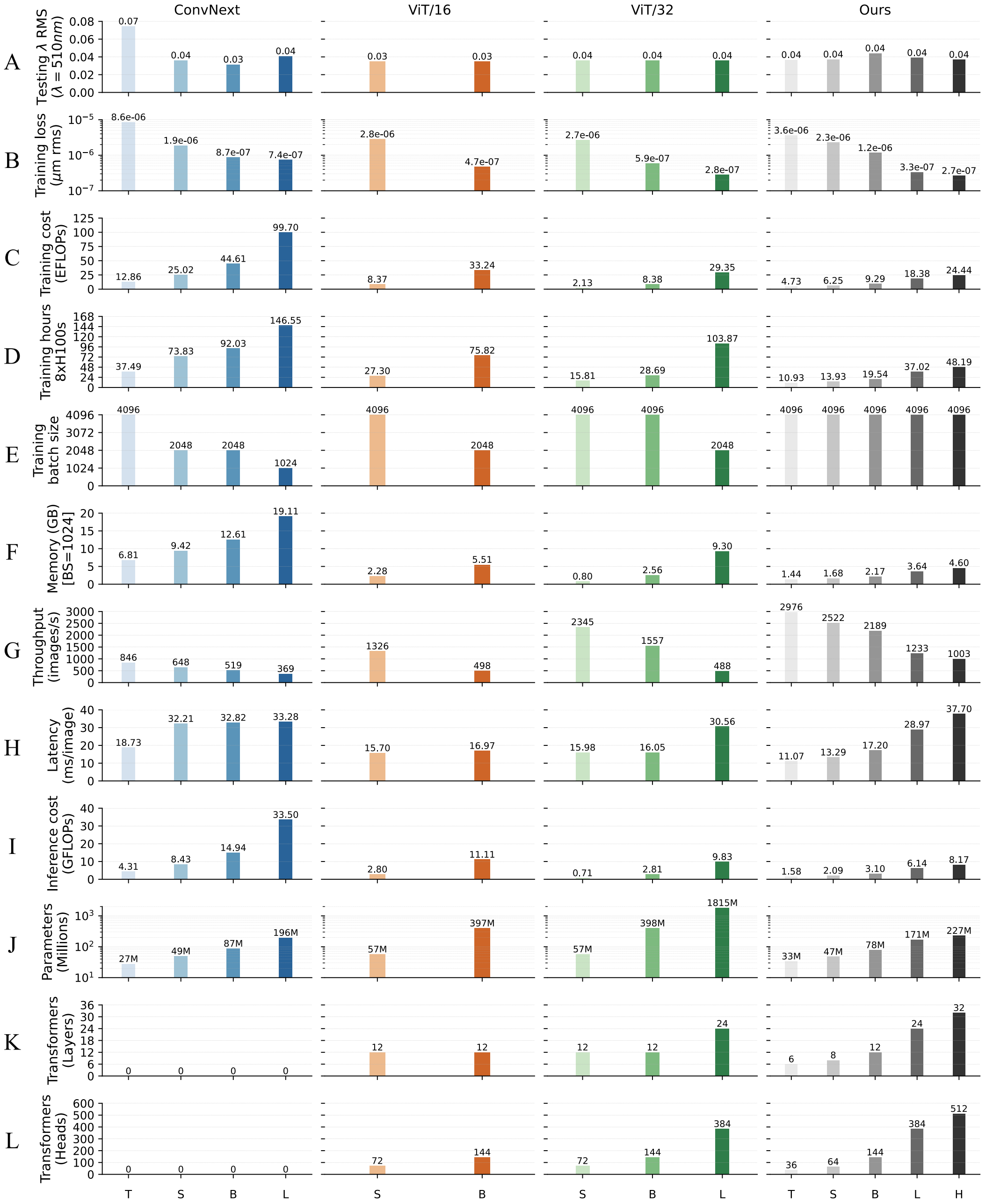

Figure S8: Comparison of the current state-of-the-art architectures applied to 3D aberration sensing across cost indicators of model efficiency. A. Median λ RMS residuals over 10K test samples after one correction for aberrations ranging between 0.1λ to 0.2λ simulated with 50K and up to 200K integrated-photons. **B.** Training loss over a dataset consisting of 2M synthetically generated samples. **C.** Training cost measured in exaFLOPS (10^{18} FLOPs) for 500 epochs of training. **D.** Training time using eight H100 GPUs. **E.** Optimal batch size used for training based on memory usage for each model. **F.** Memory footprint of each model with a batch of 1024 images using 16-bit floating point precision. **G.** Max number of predictions per second using a batch size of 1024 on a single A100 GPU. **H.** Average inference time (milliseconds) per image for a batch of 1024 examples using a single A100 GPU. **I.** Inference cost per image measured in gigaFLOPs (10^9 FLOPs). **J.** Total number of trainable parameters. **K.** Total number of transformer layers (not applicable for ConvNeXt models). **L.** Total number of heads in all transformer layers (not applicable for ConvNeXt models). Lower numbers are better for all cost indicators, except for **E**, **G**, **K**, and **L**.

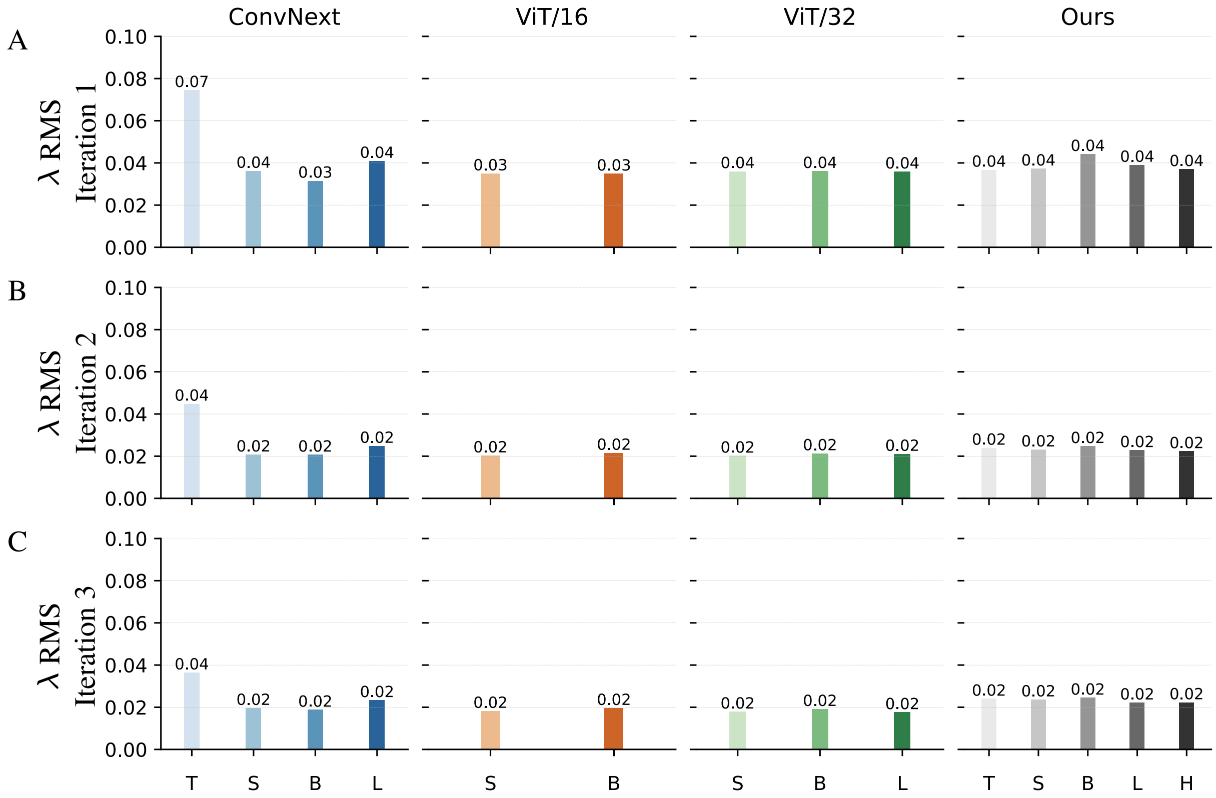

Figure S9: Median λ RMS residuals over 10K test samples with aberrations ranging between 0.2λ RMS to 0.4λ RMS, simulated with 50K and up to 200K integrated-photons. **A**. First iteration. **B**. Second iteration. **C**. Third iteration.

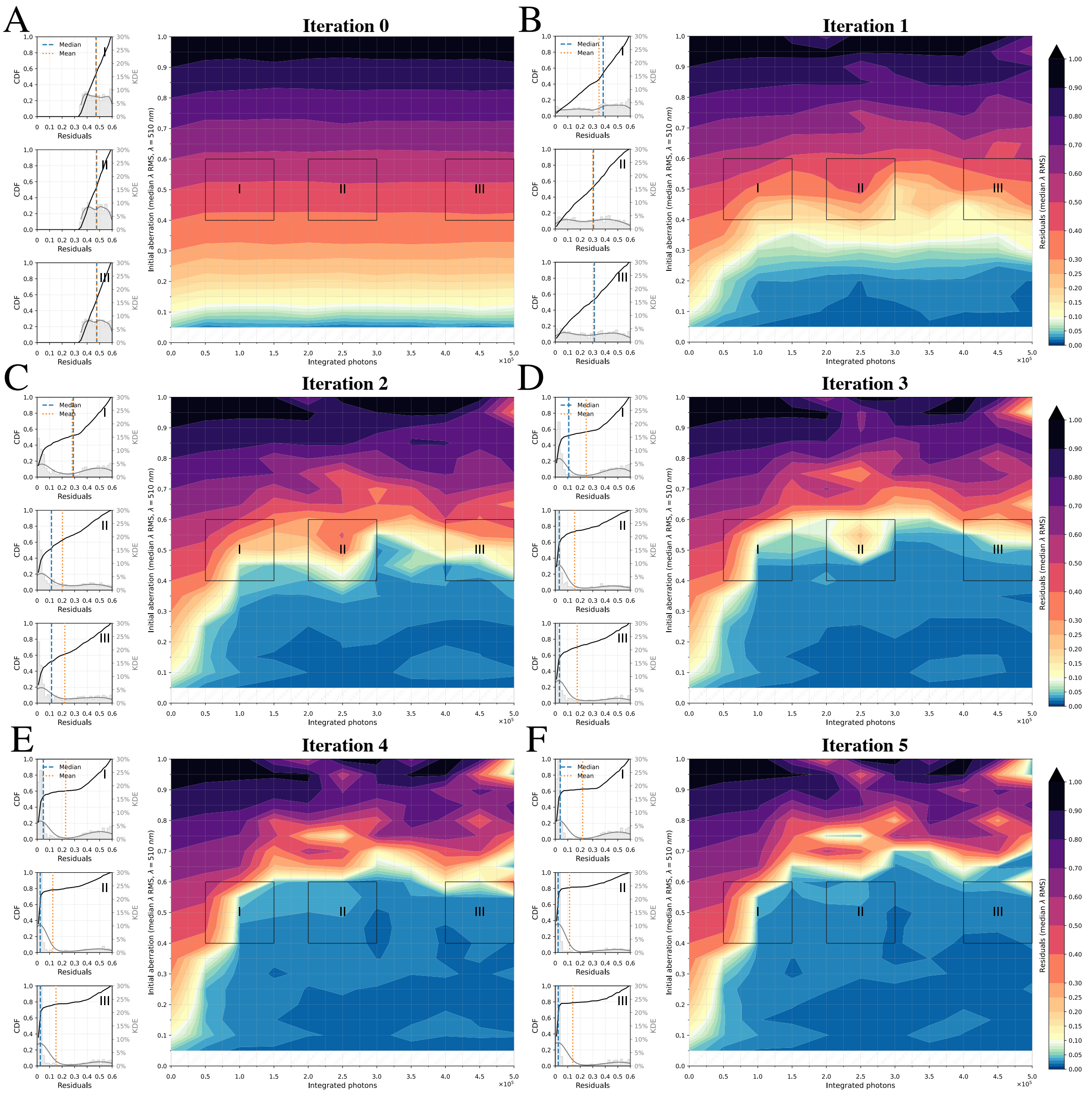

Figure S10: Sensitivity to SNR. Evaluation of our Small model (S) using simulated data of a single bead. **A**. The initial RMS distribution of test dataset as a function of SNR without AO. **B–F**. Residual λ RMS with five rounds of AO corrections.

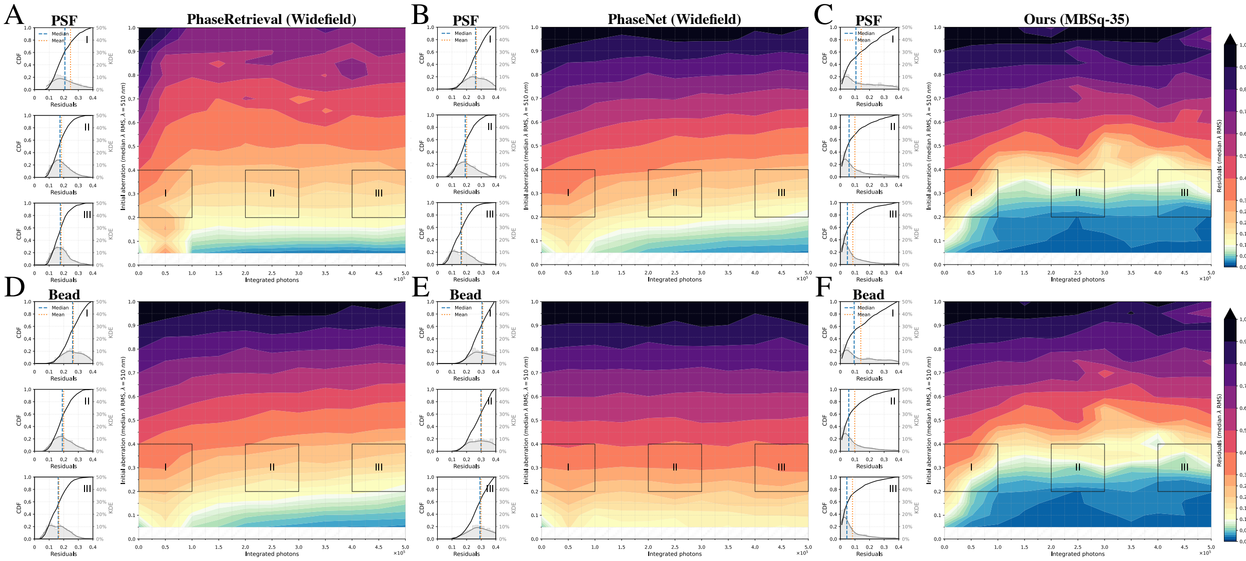

Figure S11: Singleshot evaluation of phase retrieval using 10K synthetic PSFs. A–C. Residual λ RMS for centered bead using PhaseRetrieval, PhaseNet, and our model, respectively. D–F. Residual λ RMS for a non-centered bead using the three methods mentioned above.

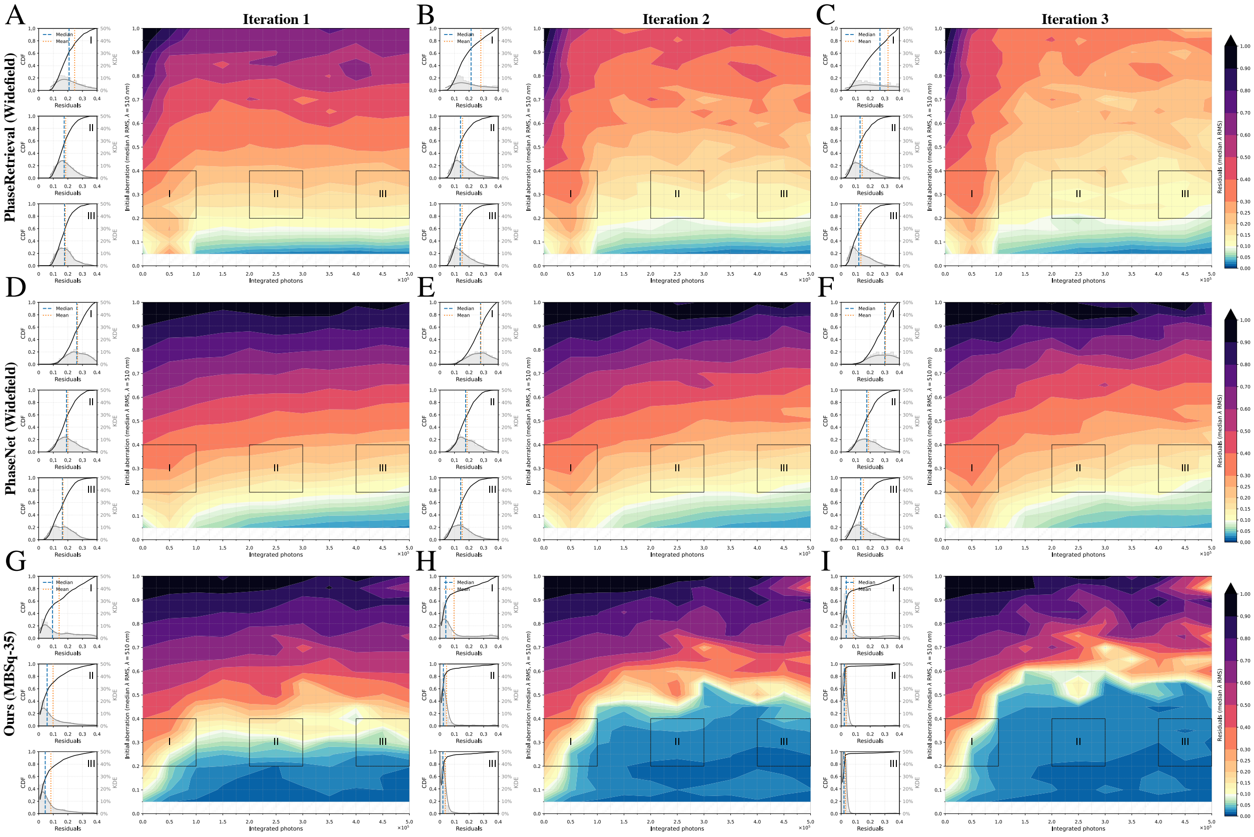

Figure S12: Iterative evaluation of phase retrieval using 10K synthetic PSFs. A–C. Residual λ RMS forwidefield PSFs using PhaseRetrieval. D–F. Residual λ RMS for widefield PSFs using PhaseNet. G–I. Residual λ RMS for YuMB LLS PSFs using our Small model (S).

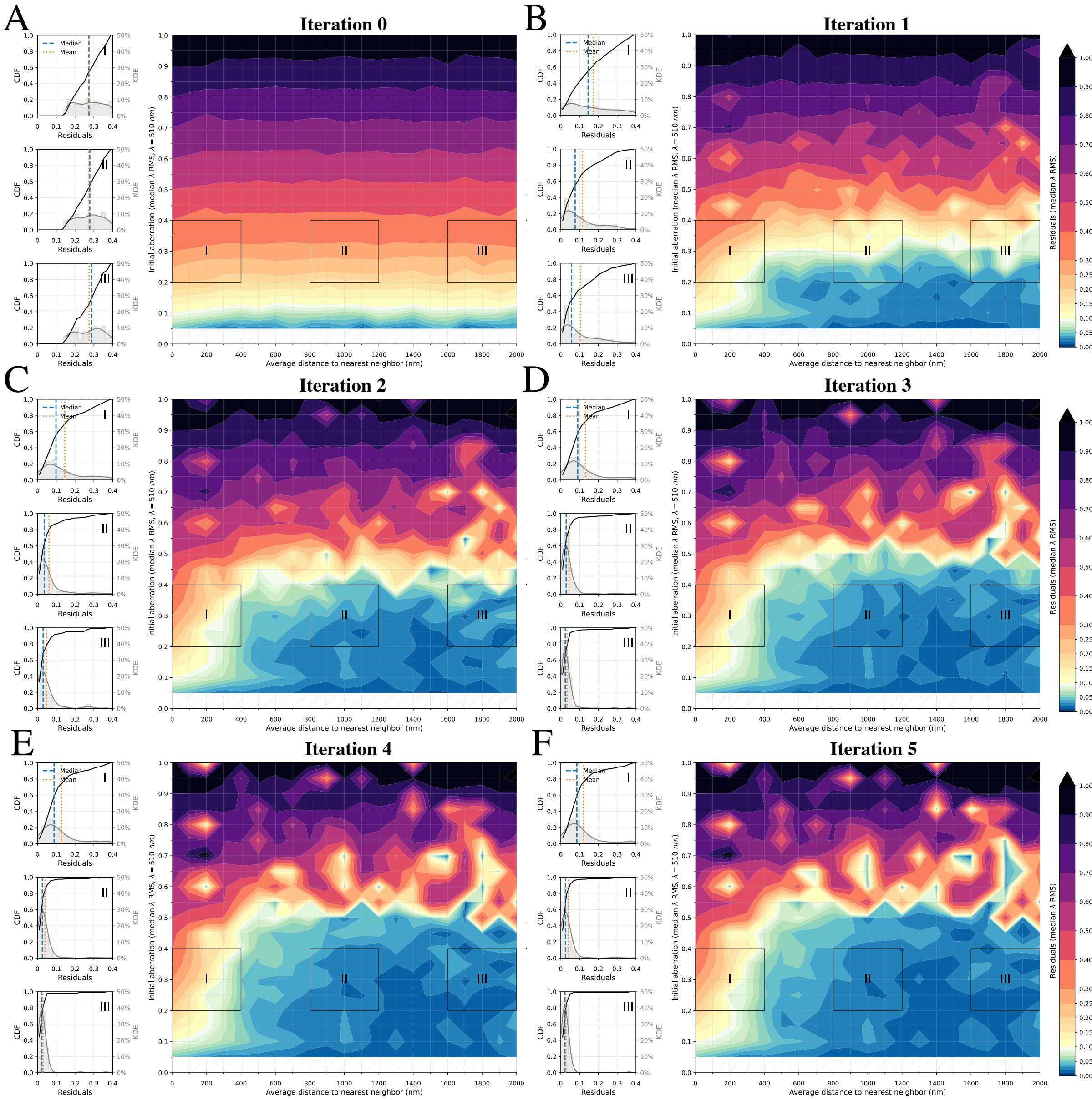

Figure S13: Sensitivity to the density of objects. Evaluation of our Small model (S) using simulated data with up to 150 beads in any given test sample. **A**. The initial RMS distribution of test dataset as a function of the average distance to the nearest neighbor (bead) without AO. **B–F**. Residual λ RMS with five rounds of AO corrections.

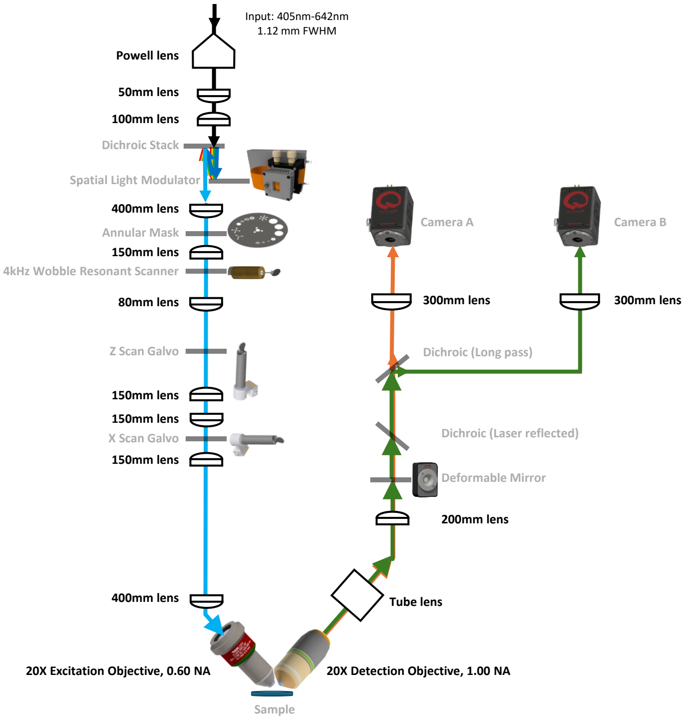

Figure S14: Schematic of AO-LLS microscope used for all experiments.

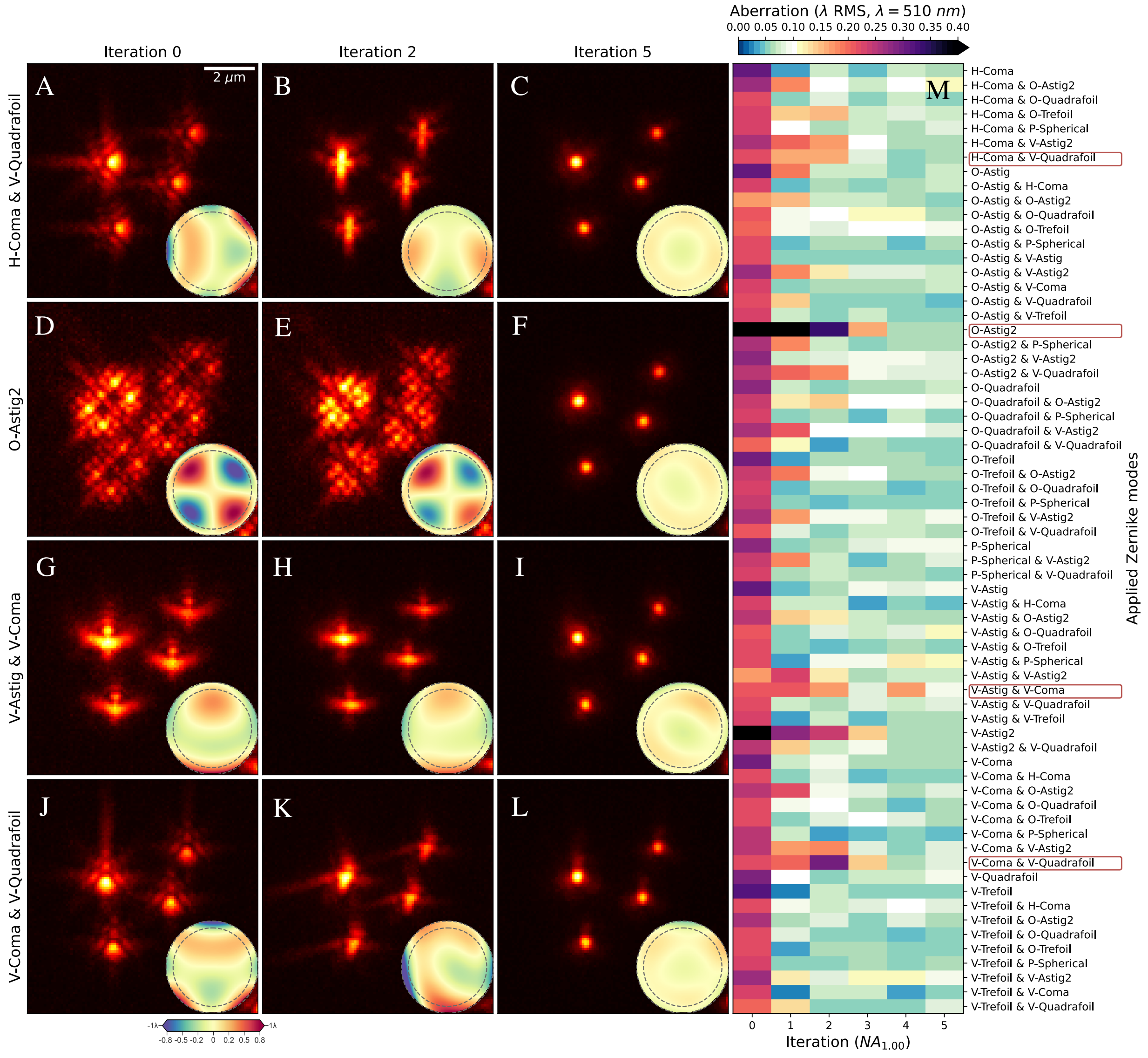

Figure S15: Correction of beads with initial artificial aberrations. Four examples, H-Coma & V-Quadrafoil ($Z_{n=3}^{m=1} + Z_{n=4}^{m=4}$), O-Astig2 ($Z_{n=4}^{m=2}$), V-Astig & V-Coma ($Z_{n=2}^{m=2} + Z_{n=3}^{m=-1}$), V-Coma & V-Quadrafoil ($Z_{n=3}^{m=-1} + Z_{n=4}^{m=4}$), where the initial aberration is artificially applied by the DM. *Iteration 0* shows XY maximum projection of four beads with initial aberration imaged using LLS, upon which AOVIFT makes predictions. *Iteration 2* shows the resulting field of beads after applying AOVIFT prediction to the DM. *Iteration 5* shows the results after applying the AOVIFT prediction measured from *Iteration 4*. Insets show the AOVIFT predicted wavefront over the NA = 1.0 pupil with a dashed line at $NA = 0.85$ **M**. Heatmap of the residual aberration (as measured via phase retrieval on a well isolated bead) after application of AOVIFT predictions starting with a single Zernike mode up to Mode 14 ($Z_{n=4}^{m=4}$) for up to 5 iterations.

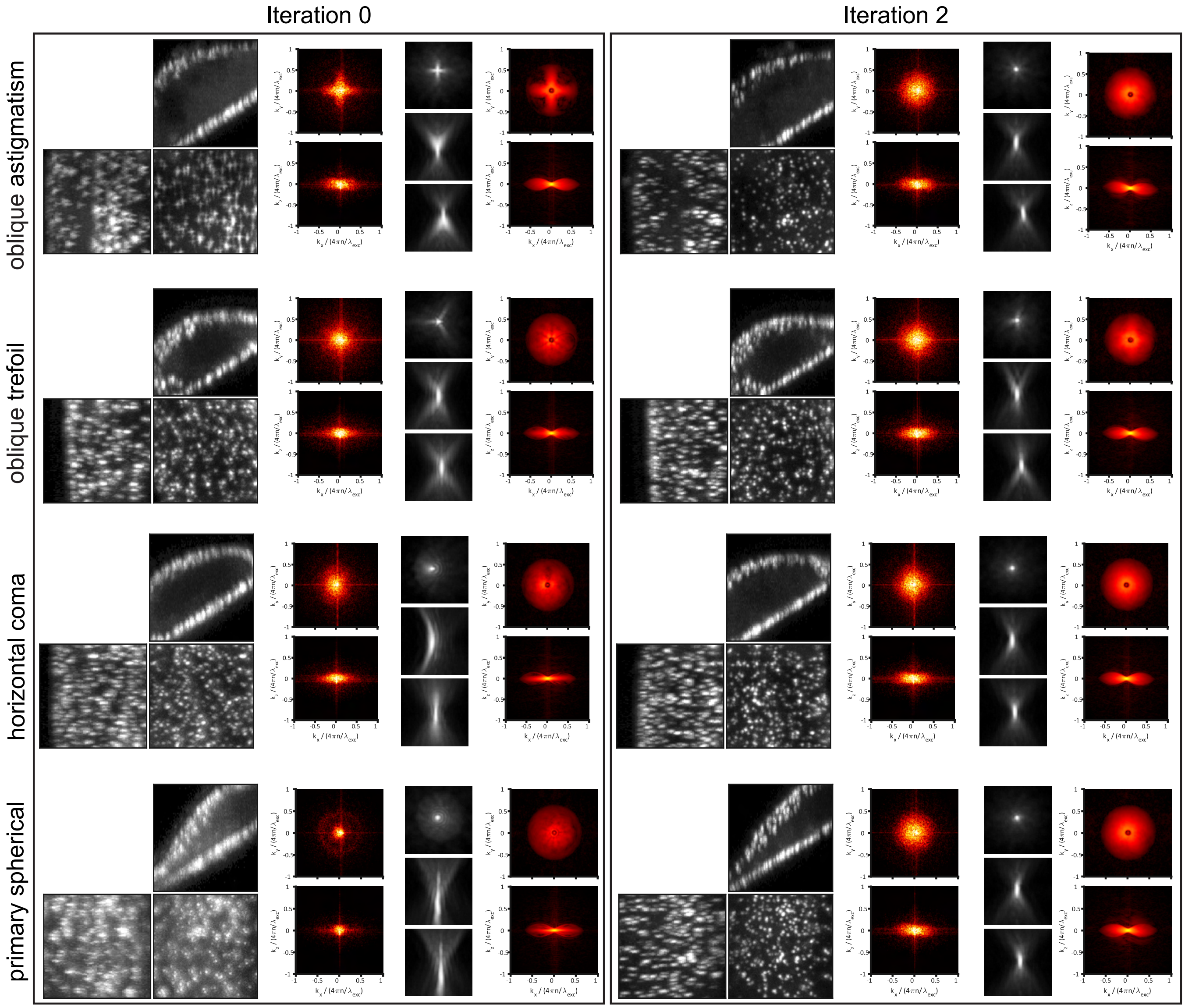

Figure S16: Experimental evaluation of AOViFT on live SUM159 AP2 cells under single mode aberrations. Four different single-mode aberrations (top to bottom) were introduced via the microscope DM to test AOViFT performance on live cells. Left panels show XY, XZ, YZ MIPs of a center-cropped region of cells; middle panels show the corresponding FFT of the cell volumes; and right panels show the widefield PSF and OTF MIPs. Each box depicts the cells, cell volume FFTs, and widefield PSF/ OTF pairs with applied single mode aberrations (Iteration 0), and after two rounds of iterative AOViFT correction (Iteration 2).

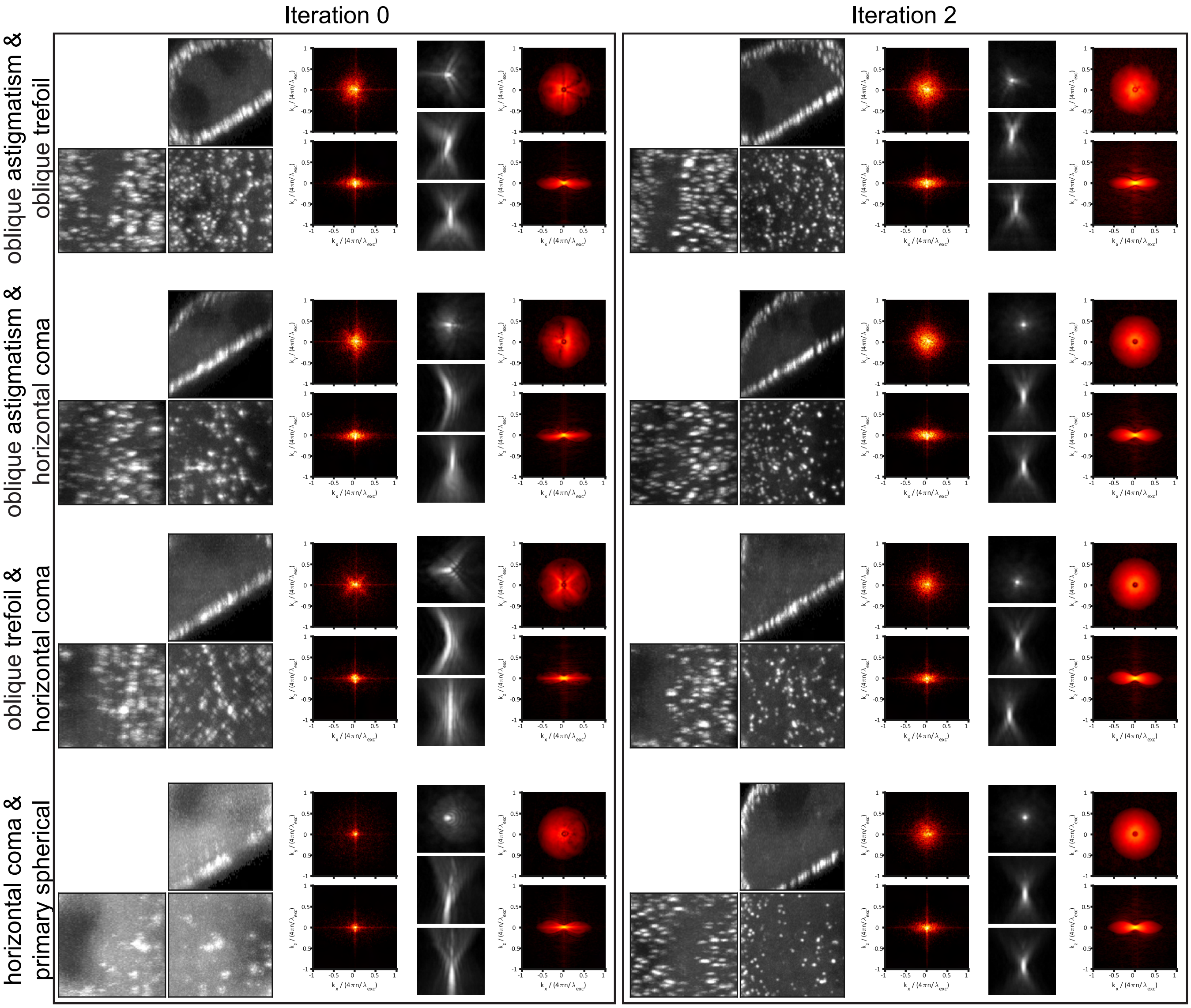

Figure S17: Experimental evaluation of AOViFT on live SUM159 AP2 cells under multiple mode aberrations. Four different two-mode aberration (top to bottom) were introduced via the microscope DM to test AOViFT performance on live cells. Left panels show XY, XZ, YZ MIPs of a center-cropped region of cells; middle panels show the corresponding FFT of the cell volumes; and right panels show the widefield PSF and OTF MIPs. Each box depicts the cells, cell volume FFTs, and widefield PSF/ OTF pairs with applied single mode aberrations (Iteration 0), and after two rounds of iterative AOViFT correction (Iteration 2)

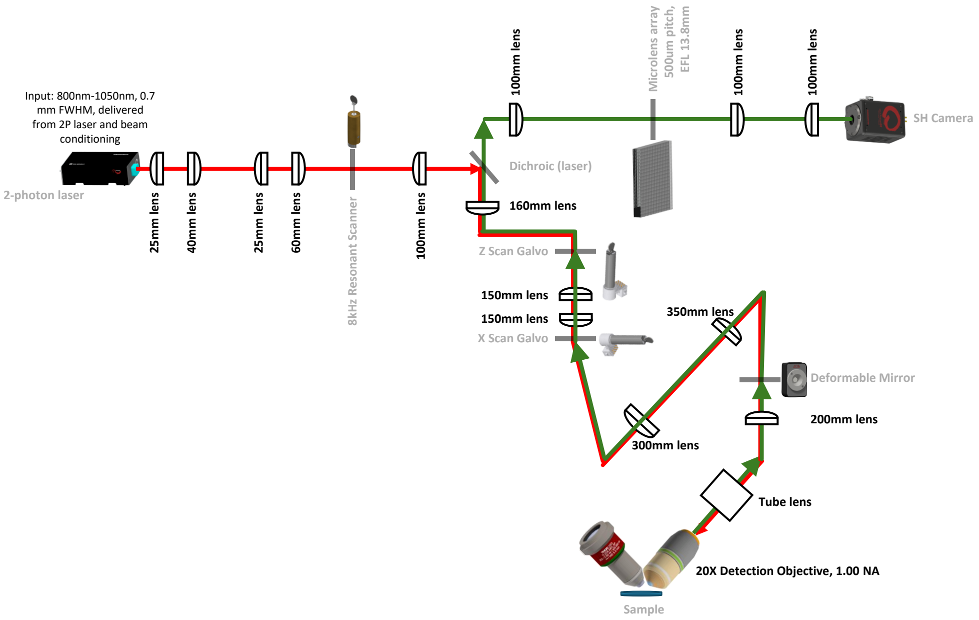

Figure S18: AO-LLS microscope schematic illustrating the Shack-Hartmann wavefront detection mode. Lenses are in 4f configuration. The X Scan Galvo, Z Scan Galvo, Annular Mask, and Deformable Mirror (DM) are at pupil conjugate planes. The Spatial Light Modulator (SLM), 4kHz wobble resonant scanner, and cameras are all at sample conjugate planes.

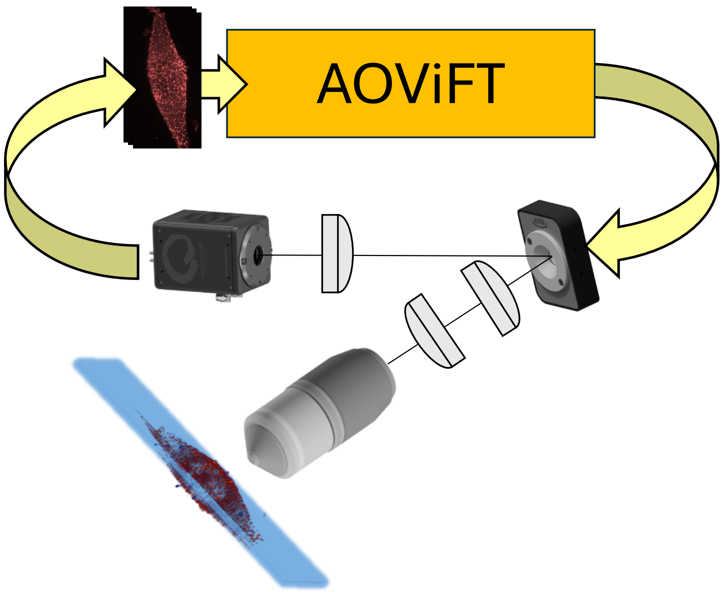

Figure S19: AOVIFT closed loop operation. Light-sheet excitation (blue), illuminates the focal plane of the detection objective causing emission from fluorescent markers (red) in the specimen. This emitted light is collected by the detection objective, relayed to a deformable mirror, and recorded by a camera. The acquired image stack is fed into AOVIFT which produces an update for the deformable mirror shape.

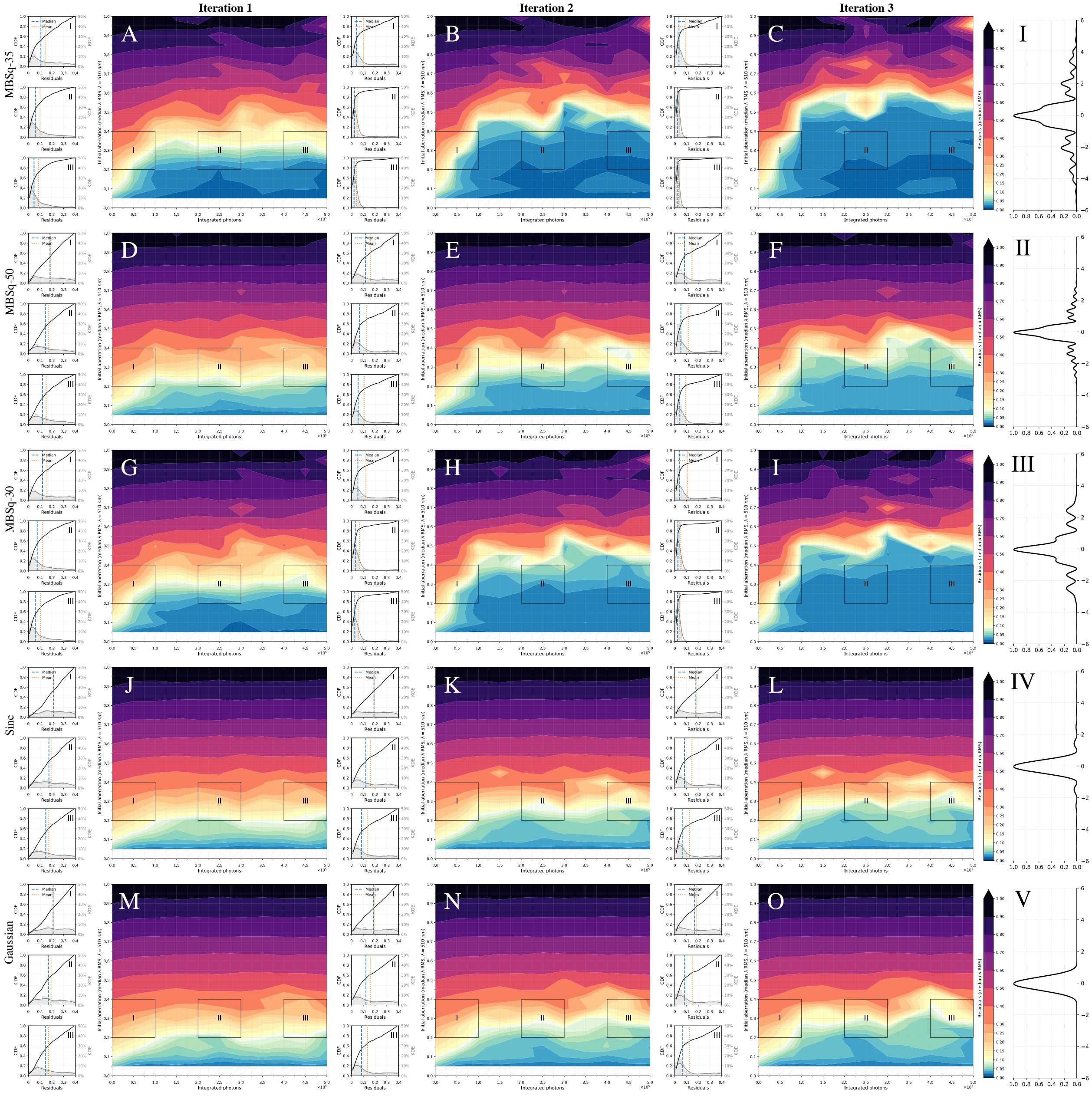

Figure S20: Evaluation of our Small model (S) using several different light sheet types over 10K synthetic samples of a single bead with aberrations up to 1.0λ RMS. **A–C**. MBSq-35 (the original LLS profile we used to simulate the training data). **D–F**. MBSq-50. **G–I**. MBSq-30. **J–L**. Sinc light sheet simulated by swept lateral standing wave. **M–O**. Gaussian light sheet. **I–V**. Excitation profiles. See Supplementary Table S6 for more details about the light sheets used for this test.

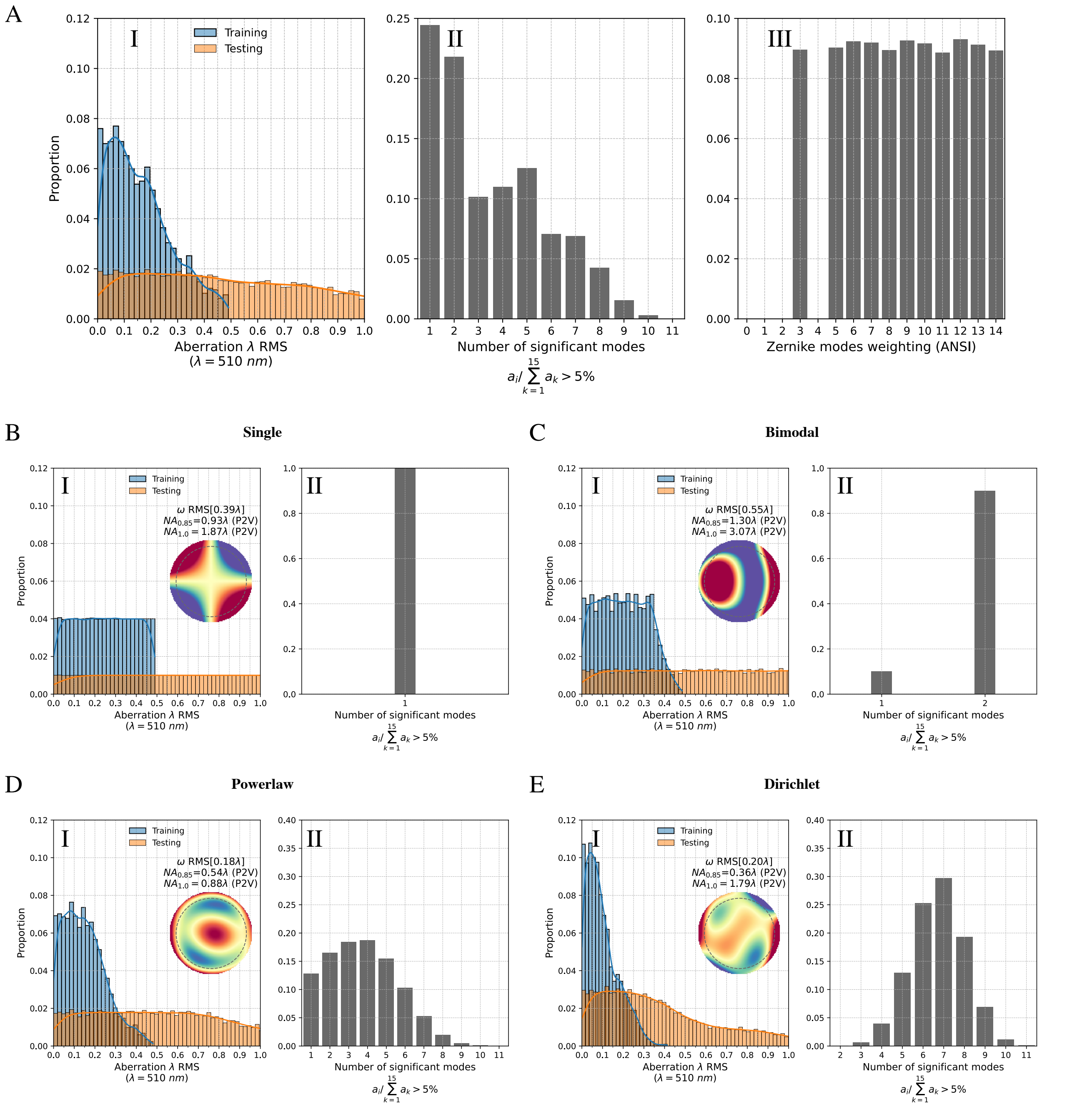

Figure S21: Zernike distributions. **A**. Statistics for the overall training and testing with four different mode distributions: single, bimodal, powerlaw, and Dirichlet. **A(I)**. Magnitude histogram for training (blue), and testing (orange) distributions. **A(II)**. A histogram showing the number of modes that contribute more than 5% of the total aberration for any given wavefornt (i.e., dominant modes). **A(III)**. A histogram illustrating the probability of choosing any of the first 15 Zernike modes. **B**. Histograms for the single mode distribution along with example wavefronts. **C**. Same, but for the bimodal distribution. **D**. Same, powerlaw distribution. **E**. Same, dirichlet distribution.

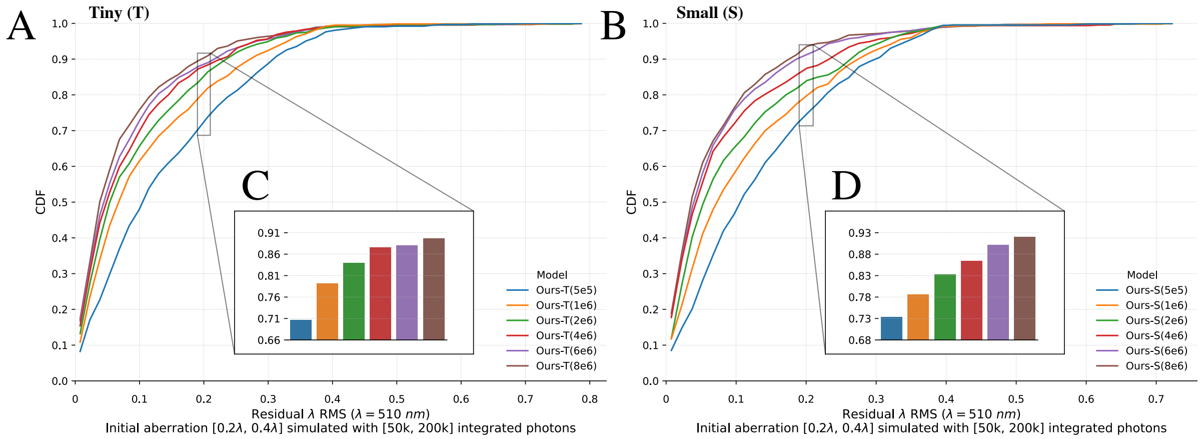

Figure S22: Scaling performance as a function of training dataset size. Residual RMS for a series of models trained with an increasing number of training samples: 500K (blue), 1M (orange), 2M (green), 4M (red), 6M (purple), and 8M (brown). To evaluate our models we show our residuals after a single correction over a test dataset of 10K samples with aberration ranging between 0.2λ RMS and 0.4λ RMS simulated using a single bead with 50K up to 200K integrated photons. **A–B**. Cumulative distribution functions for our *Tiny* model (**A**), and our *Small* model (**B**). **C–D**. Inset plots showing the percentage of data below 0.2λ RMS for each model.

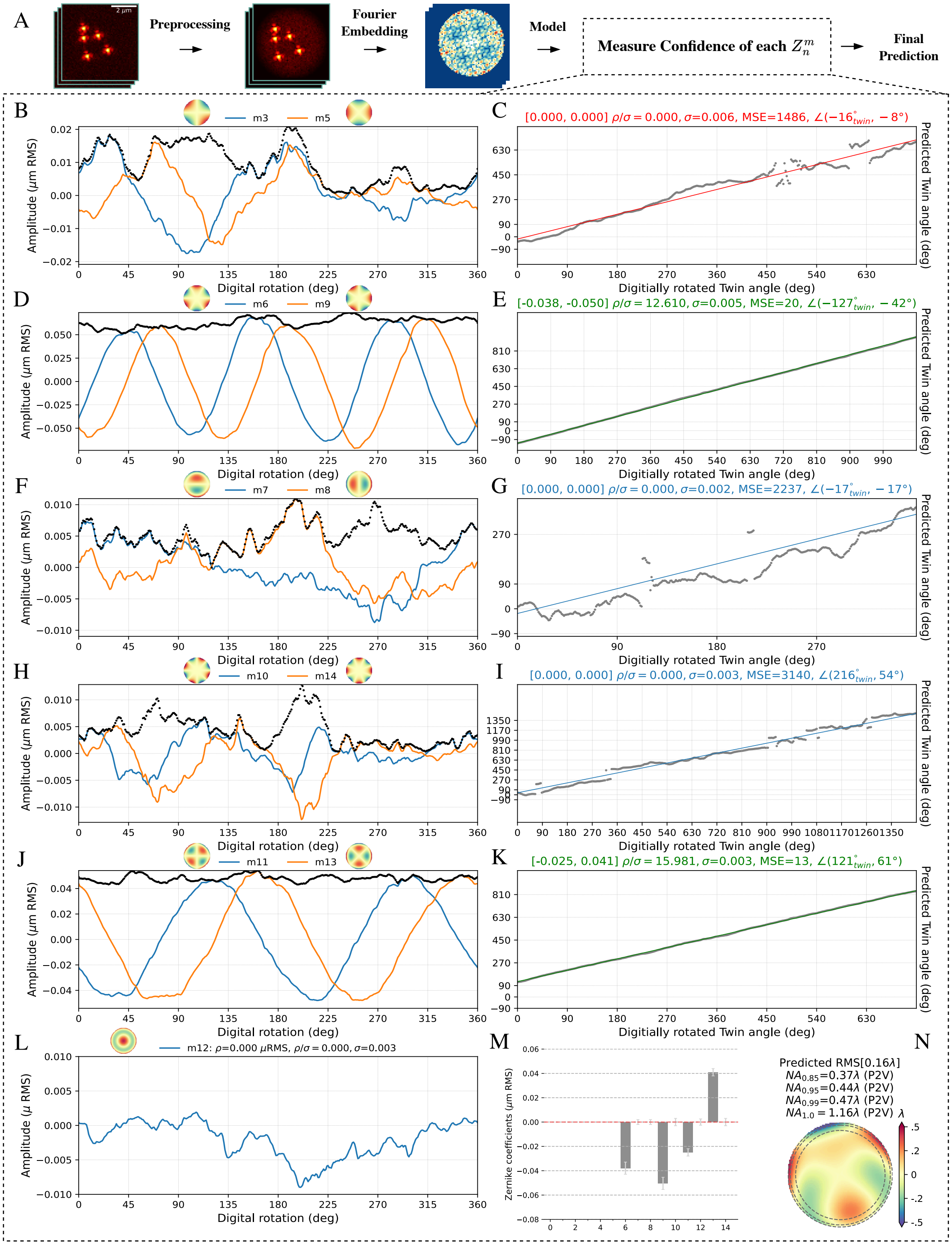

Figure S23: Predictions on rotated embeddings gives measure of confidence. **A**. Schematic of our prediction pipeline. **B**. Predicted amplitudes of astigmatism for each digital rotation: ($Z_{n=2}^{m=-2}$, blue), ($Z_{n=2}^{m=2}$, orange), and the magnitude for both twin modes in black. **C**. Regression fit between the digitally rotated angle and the predicted twin angle for astigmatism. **D–E**. Trefoil. **F–G**. Coma. **H–I**. Quadrafoil. **J–K**. Secondary astigmatism. **L**. Predicted amplitudes of primary spherical for each digital rotation. **M**. Final prediction for each Zernike mode. **N**. Predicted wavefront.

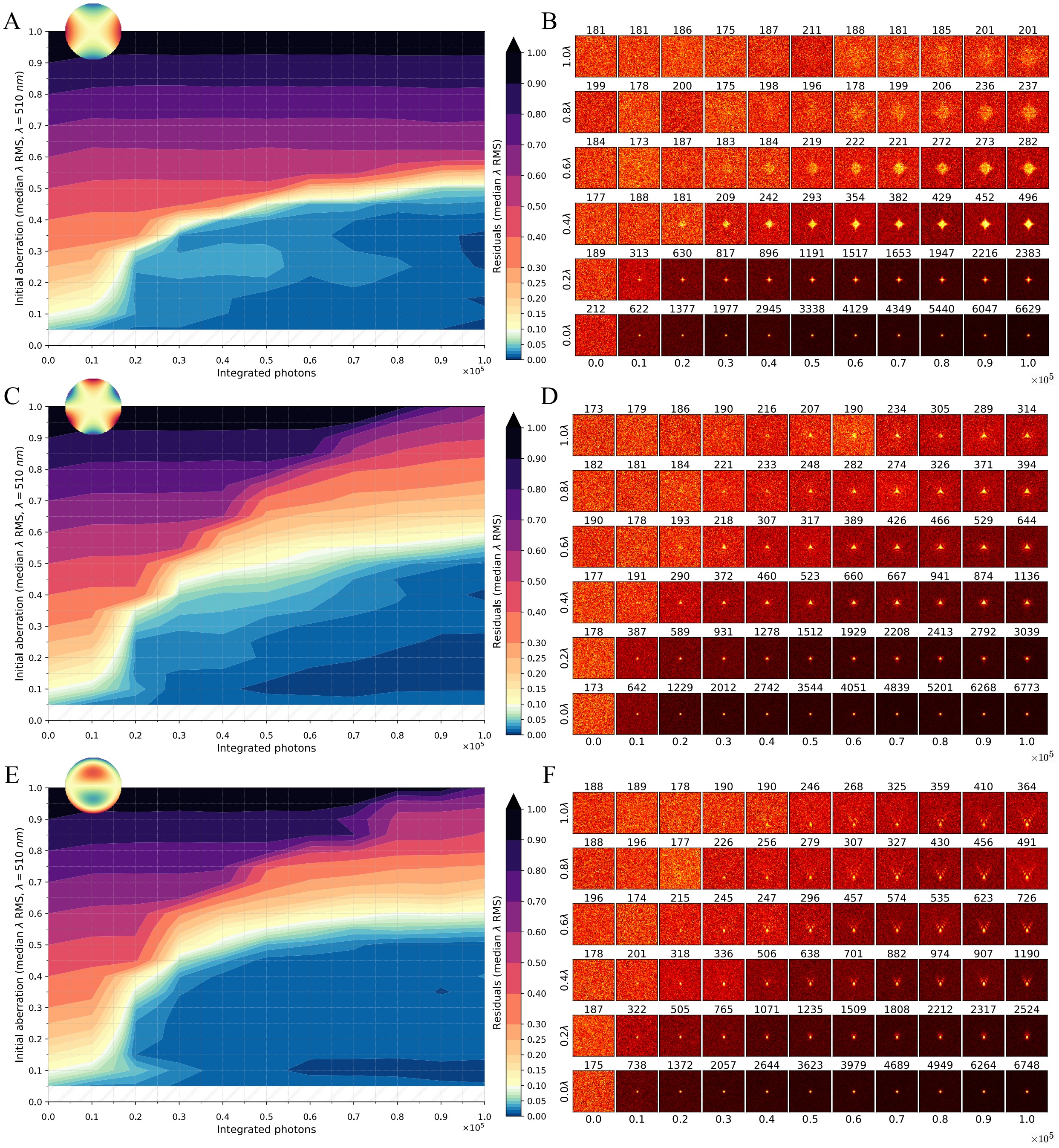

Figure S24: Single mode evaluation (I). **A**. Residual λ RMS after a single correction using our Small model (S) for vertical astigmatism $Z_{n=2}^{m=2}$. **B**. XY MIPs showing the initial aberration without correction for different amplitudes *w.r.t.* SNR, with the max counts highlighted above each PSF. **C–D**. Vertical trefoil $Z_{n=3}^{m=-3}$. **E–F**. Vertical coma $Z_{n=3}^{m=-1}$.

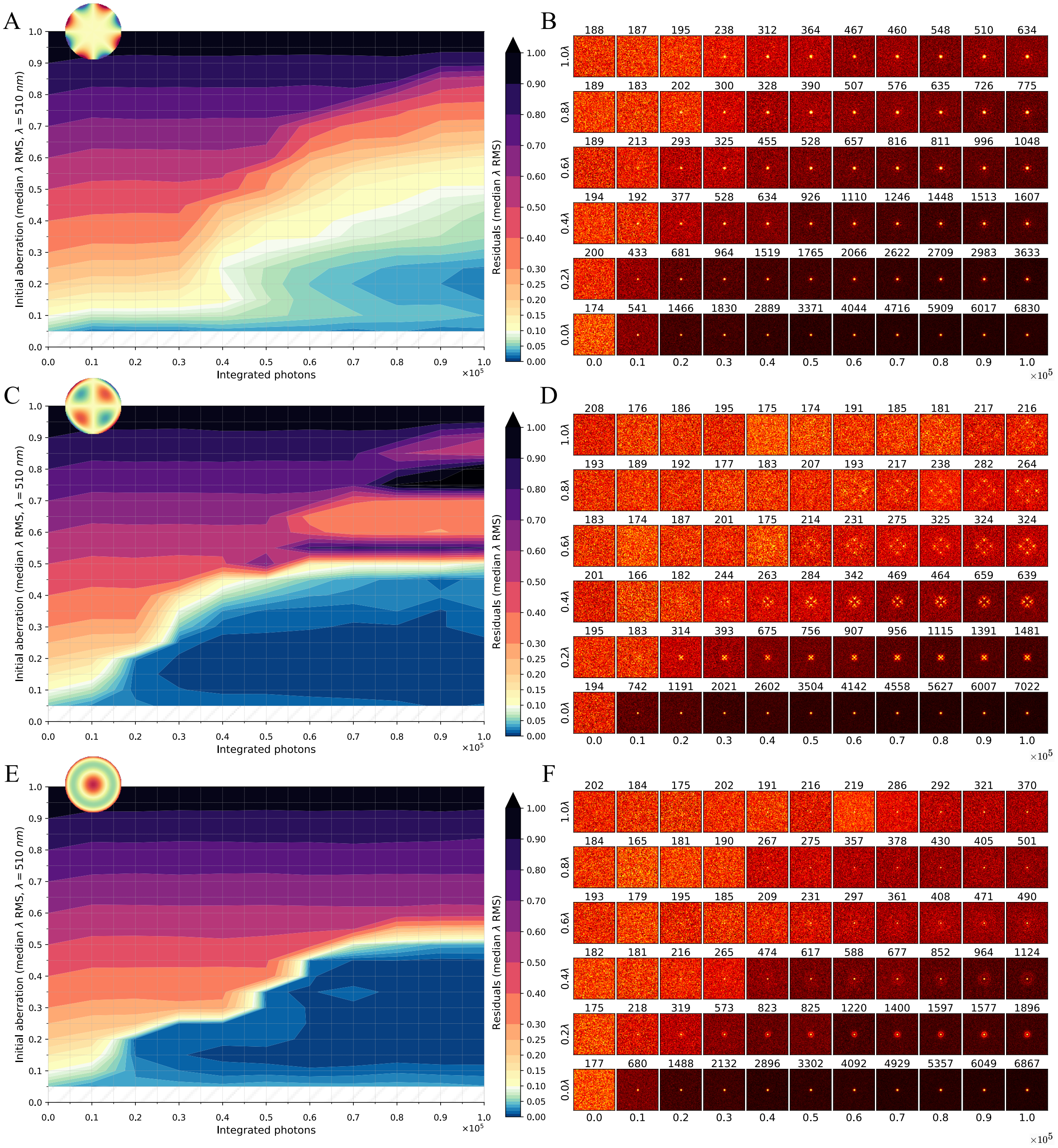

Figure S25: Single mode evaluation (II). **A**. Residual λ RMS after a single correction using our Small model (S) for oblique quadrafoil $Z_{n=4}^{m=-4}$. **B**. XY MIPs showing the intitial aberration without correction for different amplitudes *w.r.t.* SNR, with the max counts highlighted above each PSF. **C–D**. Oblique secondary astigmatism $Z_{n=4}^{m=-2}$. **E–F**. Primary spherical $Z_{n=4}^{m=0}$.

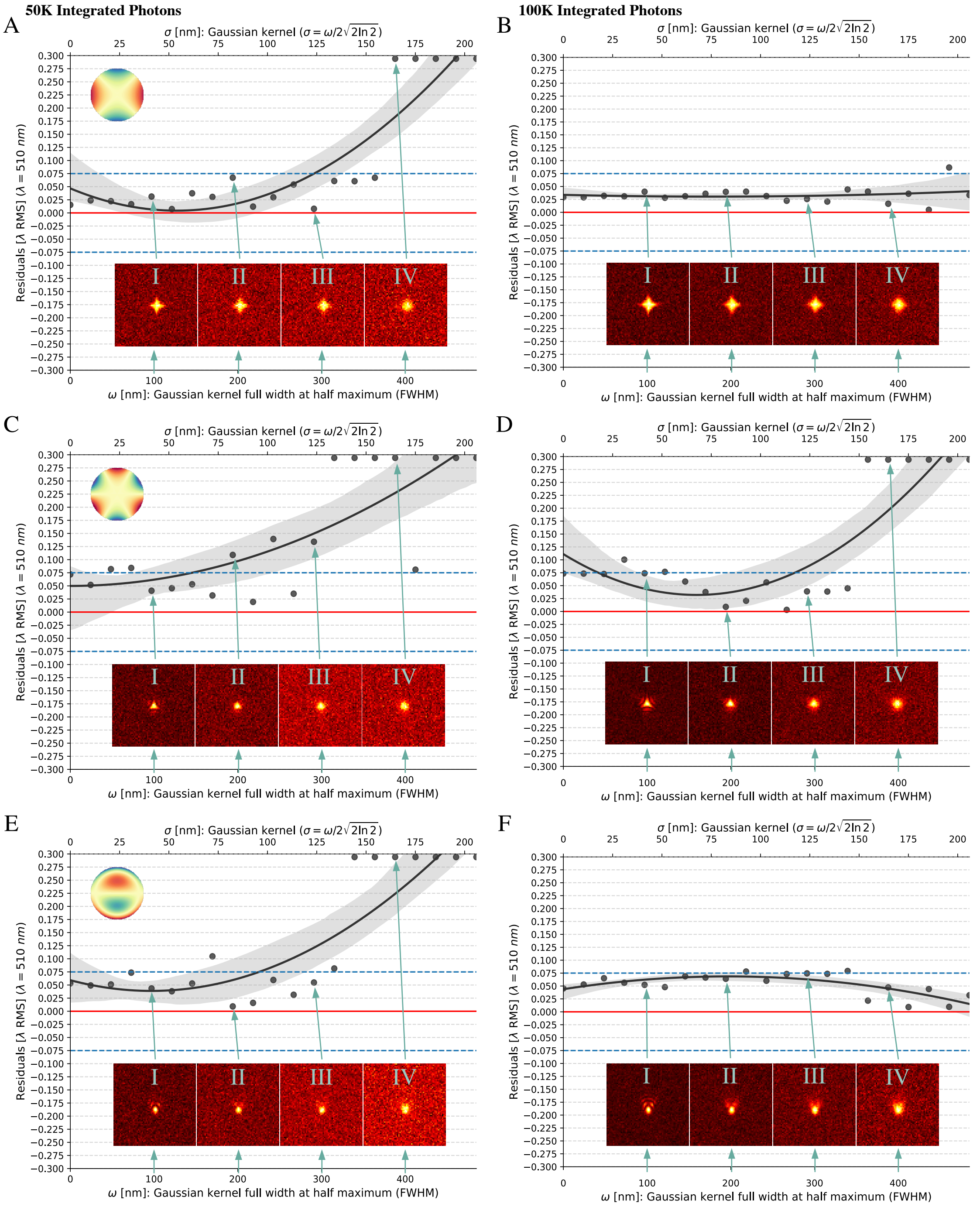

Figure S26: Sensitivity to object size. We use 0.3 λ RMS to synthetically generate a single mode aberration, while increasing the size of the Gaussian kernel used to simulate the bead for each sample. **A**. Residual λ RMS after a single correction using our Small model (S) for vertical astigmatism $Z_{n=2}^{m=2}$ simulated with 50K integrated photons. **B**. Residual λ RMS using PSFs simulated with 100K integrated photons. **C–D**. Residual λ RMS for vertical trefoil $Z_{n=3}^{m=-3}$. **E–F**. Residual λ RMS for vertical coma $Z_{n=3}^{m=-1}$.

Table S1: Comparisons with other ML-based AO methods.

Paper	**Speed**<br>(time/isoplanatic patch)	**Accuracy and Robustness**	**Noninvasiveness**<br>(photobleaching)	**Code Available?**
[4]	≤ 1s.	3D widefield PSFs.<br>(Fig. S11–S12)	No extra imaging.	✓
[5]	≤ 5s.	2D data.<br>Samples that are sparse,<br>cleared, fixed, and high SNR.	1 extra (defocused) image.	✗
[6]	≤ 1s.	3D data.<br>Single Molecule Light Microscopy<br>data only with small aberration.<br>Kalman filter needed for robustness.	No extra imaging.	✓
[7]	Extra time needed<br>to acquire M more extra images<br>(M=2,4 or 2N, 4N).	2D data.<br>Diverse sample types.<br>Many manually-tuned parameters.<br>Best at 0.15–0.3 λ RMS.<br>Less accurate at 0.1–0.13 λ RMS.	Many extra images<br>and motion correction<br>recommended.	✗
[8]	~5 minuets per isoplanatic<br>patch of 32 megavoxels<br>(200×400×400).	3D data.<br>Local thresholding required<br>to remove hallucinations.<br>Tested with beads and<br>sparse neuronal structures.<br>Aberration up to 0.3 λ RMS.<br>Many manually-tuned parameters.	No extra imaging.	✓
Ours	≤ 3s (Table. S5).	3D data with puncta.<br>Aberration up to 1.0 λ RMS<br>(Appendix C, Fig. S20).<br>Confidence scores<br>(Appendix A.6).	No extra imaging.	✓

Table S2: Hyperparameters for our model variants. A breakdown of the hyperparameters used for each stage i, where p_i is the patch size used to tile in the input tensor, n_i is the number of transformer layers, h_i is the number of heads for each transformer layer, ϵ_i is the embedding size, and x_i is the MLP size. Furthermore, we show the total number of trainable parameters based on the scheme used to derive each variant with the total number of transformer layers used across all stages (n) and the number of heads for each transformer layer (h).

	**Stage** 1					**Stage** 2					**Scheme**		**Params**
**Name**	Patch p_1	Layers n_1	Heads h_1	EMB ϵ_1	MLP x_1	Patch p_2	Layers n_2	Heads h_2	EMB ϵ_2	MLP x_2	Layers n	Heads h	millions
**Tiny (T)**	32	3	6	1024	4096	16	3	6	256	1024	6	6	33.9
**Small (S)**	32	4	8	1024	4096	16	4	8	256	1024	8	8	47.4
**Base (B)**	32	6	12	1024	4096	16	6	12	256	1024	12	12	78.3
**Large (L)**	32	12	16	1024	4096	16	12	16	256	1024	24	16	171.3
**Huge (H)**	32	16	16	1024	4096	16	16	16	256	1024	32	16	227.9

Table S3: Evaluation benchmark.

		Testing λ RMS (one shot)	(two shot)↓	Loss (μm RMS)↓	Training hours↓	Training EFLOPs↓	Memory (GB)↓	Throughput (img/s)↑	Latency (ms/img)↓	Inference GFLOPs↓	Parameters (millions)↓
ConvNext	**T**	0.07	0.04	$85.6e^{-7}$	37.5	12.9	6.8	846	18.7	4.3	27.9
	**S**	0.04	0.02	$18.8e^{-7}$	73.8	25.0	9.4	648	32.2	8.4	49.6
	**B**	0.03	0.02	$8.7e^{-7}$	92.0	44.6	12.6	519	32.8	14.9	87.7
	**L**	0.04	0.02	$7.4e^{-7}$	146.6	99.7	19.1	369	33.3	33.5	196.4
ViT/16	**S**	0.03	0.02	$28.4e^{-7}$	27.3	8.4	2.3	1326	15.7	2.8	71.2
	**B**	0.03	0.02	$4.7e^{-7}$	75.8	33.2	5.5	498	17.0	11.1	397.6
ViT/32	**S**	0.04	0.02	$26.8e^{-7}$	15.8	2.1	0.9	2345	16.0	0.7	71.5
	**B**	0.04	0.02	$5.9e^{-7}$	28.7	8.4	2.6	1557	16.1	2.8	398.2
	**L**	0.04	0.02	$2.8e^{-7}$	103.9	29.4	9.3	488	30.6	9.8	1815.0
Ours	**T**	0.04	0.02	$36.3e^{-7}$	10.9	4.7	1.4	2976	11.1	1.6	33.9
	**S**	0.04	0.02	$22.9e^{-7}$	13.9	6.3	1.7	2522	13.3	2.1	47.4
	**B**	0.04	0.02	$11.7e^{-7}$	19.5	9.3	2.2	2189	17.2	3.1	78.3
	**L**	0.04	0.02	$3.3e^{-7}$	37.0	18.4	3.6	1233	29.0	6.1	171.3
	**H**	0.04	0.02	$2.7e^{-7}$	48.2	24.4	4.6	1003	37.7	8.2	227.9

Table S4: Residual wavefront measured using phase retrieval with widefield on an isolated bead for Fig. 4.

**Iteration**	**NA**	**Residual WF** λ RMS	**Residual WF** λ P2V
		**Fig. 4a**	
0	1.00	0.450701	2.940459
0	0.95	0.380205	2.425910
0	0.85	0.292638	1.435171
1	1.00	0.155836	1.032606
1	0.95	0.135578	0.927065
1	0.85	0.096931	0.705702
2	1.00	0.089000	0.479712
2	0.95	0.083749	0.370095
2	0.85	0.077435	0.336847
		**Fig. 4b**	
0	1.00	0.536977	3.053908
0	0.95	0.465103	2.359822
0	0.85	0.425599	1.941650
1	1.00	0.071351	0.459885
1	0.95	0.061207	0.330569
1	0.85	0.054622	0.244614
2	1.00	0.068686	0.459620
2	0.95	0.059220	0.362715
2	0.85	0.048254	0.218788

Table S5: Performance of SH and AOVIFT-S running on a single node with either one or four A100 GPUs. Benchmark for three modes of operations: default mode, smart ROI detection mode where a ROI containing diffraction-limited puncta are cropped from a scanned volume, and tiling mode. AOVIFT-S measurement is done on the entire 3D volume (z × y × x μm^3), whereas SH measurement is done on 3 (y × x μm^2) planes. Total time includes initialization and setup time (e.g., loading model onto each GPU), which may take between 5 and 10 seconds but is only needed at the very beginning of an imaging experiment. Since aberration measurement by SH is done serially, the SH [tile] values reported here represent the average measurement time for 288 ROIs based on the SH [default] measurement time for a single ROI. We also show a breakdown of the time needed for preprocessing & embedding, smart ROI detection, and model inference time.

**Method**	**Predictions** w/ 361 rotations	**Preprocessing Time**	**ROI Detection Time**	**Inference Time**	**Total Time**	**GPUs** (A100)
**Beads** 12.8 × 9.3 × 9.3 μm^3						
AOVIFT-S [default]	1	2s	N/A	3s	8.6s	1
AOVIFT-S [default]	361	2s	N/A	3s	9.2s	1
AOVIFT-S [default]	361	2s	N/A	9s	16.9s	4
**Cells** 25.6 × 15.5 × 55.9 μm^3						(ROI = 12.8, 6.2, 6.2 μm^3)
AOVIFT-S [default]	1	3s	N/A	3s	8.9s	1
AOVIFT-S [default]	361	3s	N/A	3s	9.3s	1
AOVIFT-S [default]	361	3s	N/A	9s	18.1s	4
AOVIFT-S [roi-1]	361	3s	3s	9s	25.3s	4
AOVIFT-S [roi-10]	3610	6s	9s	15s	40.6s	4
**Zebrafish small FOV** 12.8 × 12.4 × 12.4 μm^3						(ROI = 12.8, 6.2, 6.2 μm^3)
SH [default]	1	N/A	N/A	N/A	5.1s	N/A
AOVIFT-S [default]	1	3s	N/A	3s	8.8s	1
AOVIFT-S [default]	361	3s	N/A	3s	9.2s	1
AOVIFT-S [default]	361	3s	N/A	9s	17.0s	4
AOVIFT-S [roi-1]	361	3s	3s	9s	23.7s	4
AOVIFT-S [roi-10]	3610	6s	9s	15s	38.9s	4
**Zebrafish large FOV** 12.8 × 49.7 × 223.5 μm^3						(TILE = 12.8, 9.3, 9.3 μm^3)
SH [tile]	288 tiles	N/A	N/A	N/A	1,468.8s	N/A
AOVIFT-S [tile]	103,968 (288 tiles × 361)	30.6s	N/A	91.4s	139.8s	4

Table S6: Light sheet specifications. *MBSq-35* LLS excitation profile was used for simulating training data.

Name	Lattice type	$NA_{lattice}$	NA_{exc}	NA_{sinc}	$NA_{annulus}$	σ_{NA}	FWHM	Crop
**MBSq-30**	Multi-Bessel square LLS	0.30	-	-	0.375/0.225	0.10	48.5	-
**MBSq-35**	Multi-Bessel square LLS	0.35	-	-	0.40/0.30	0.10	-	-
**MBSq-50**	Multi-Bessel square LLS	0.50	-	-	0.40/0.30	0.10	-	-
**Sinc**	Simulated by swept lateral standing wave	-	0.32	0.24	0.40/0.20	-	51.5	-
**Gaussian**	Simulated by swept lateral standing wave	-	0.21	-	0.40/0.20	0.21	51.0	0.1

Table S7: Imaging configuration. All data was collected using *MBSq-35* light sheet described in Supplementary Table S6.

**Figure | Scan type**	**Voxel size** (obj x, obj y, obj z)	**Volume size [voxels]** (obj x, obj y, obj z)	**Excitation wavelength**	**Camera Exposure**
**Fig. 4** | Widefield Scan	97, 97, 100 nm	96, 96, 128	488 nm	50 ms
**Fig. 4** | Cell Scan	97, 97, 200 nm	160, 576, 128	488 nm, 560 nm	50 ms
**Fig. 5a** | No AO Full Chip Scan	97, 97, 200 nm	2304, 2304, 64	488 nm , 560 nm	50 ms
**Fig. 5b** | No AO Scan 1	97, 97, 200 nm	128, 128, 64	488 nm, 560 nm	50 ms
**Fig. 5b** | DSH 1	0.1, 6.2, 6.3 um	125, 3, 3	920 nm	500 ms
**Fig. 5b** | DSH 2	0.1, 6.2, 6.3 um	125, 3, 3	920 nm	400 ms
**Fig. 5b** | DSH 3	0.1, 6.2, 6.3 um	125, 3, 3	920 nm	400 ms
**Fig. 5b** | SH AO Scan	97, 97, 200 nm	128, 128, 64	488 nm , 560 nm	50 ms
**Fig. 5b** | No AO Scan 2	97, 97, 200 nm	128, 128, 64	488 nm , 560 nm	50 ms
**Fig. 5b** | AOViFT Iter 1 Scan	97, 97, 200 nm	128, 128, 64	488 nm , 560 nm	50 ms
**Fig. 5b** | AOViFT Iter 2 Scan	97, 97, 200 nm	128, 128, 64	488 nm , 560 nm	50 ms
**Fig. 5c** | No AO Full Chip Scan	97, 97, 200 nm	2304, 2304, 64	488 nm, 560 nm	50 ms, 50 ms
**Fig. 5c** | No AO Scan 1	97, 97, 200 nm	128, 128, 64	488 nm, 560 nm	50 ms, 50 ms
**Fig. 5c** | DSH 1	0.1, 6.2, 6.3 um	125, 3, 3	920 nm	500 ms
**Fig. 5c** | DSH 2	0.1, 6.2, 6.3 um	125, 3, 3	920 nm	500 ms
**Fig. 5c** | DSH 3	0.1, 6.2, 6.3 um	125, 3, 3	920 nm	500 ms
**Fig. 5c** | SH AO Scan	97, 97, 200 nm	128, 128, 64	488 nm, 560 nm	50 ms, 50 ms
**Fig. 5c** | No AO Scan 2	97, 97, 200 nm	128, 128, 64	488 nm, 560 nm	50 ms, 50 ms
**Fig. 5c** | AOViFT Iter 1 Scan	97, 97, 200 nm	128, 128, 64	488 nm, 560 nm	50 ms, 50 ms
**Fig. 5c** | AOViFT Iter 2 Scan	97, 97, 200 nm	128, 128, 64	488 nm, 560 nm	50 ms, 50 ms
**Fig. 5c** | AOViFT Full Chip Scan	97, 97, 200 nm	2304, 2304, 64	488 nm, 560 nm	50 ms, 50 ms
**Fig. 6b** | AOViFT Full Chip Scan	97, 97, 200 nm	2304, 2304, 64	488 nm	50 ms
**Fig. 6d** | AOViFT Full Chip Scan	97, 97, 200 nm	2304, 2304, 64	560 nm	50 ms
**Fig. S16–S17** | No AO Full Chip Scan	97, 97, 200 nm	2304, 2304, 64	488 nm, 560 nm	50 ms, 50 ms
**Fig. S16–S17** | No AO Scan 1	97, 97, 200 nm	256, 256, 64	488 nm, 560 nm	50 ms, 50 ms
**Fig. S16–S17** | DSH 1	0.1, 6.2, 6.2 um	125, 3, 3	920 nm	400 ms
**Fig. S16–S17** | DSH 2	0.1, 6.2, 6.2 um	125, 3, 3	920 nm	400 ms
**Fig. S16–S17** | DSH 3	0.1, 6.2, 6.2 um	125, 3, 3	920 nm	400 ms
**Fig. S16–S17** | SH AO Scan	97, 97, 200 nm	256, 256, 64	488 nm, 560 nm	50 ms, 50 ms
**Fig. S16–S17** | No AO Scan 2	97, 97, 200 nm	256, 256, 64	488 nm, 560 nm	50 ms, 50 ms
**Fig. S16–S17** | AOViFT Iter 1 Scan	97, 97, 200 nm	256, 256, 64	488 nm, 560 nm	50 ms, 50 ms
**Fig. S16–S17** | AOViFT Iter 2 Scan	97, 97, 200 nm	256, 256, 64	488 nm, 560 nm	50 ms, 50 ms

Table S8: Training configuration.

**Optical configuration**	
Lattice type	*MBSq-35* (Supplementary Table S6)
NA_{exc}	0.35
$NA_{annulus}$	0.4/0.3
$NA_{detection}$	1.0
σ_{NA}	0.10
Refractive index	1.33
Excitation wavelength	488 nm
Detection wavelength (λ)	510 nm
Voxel size	$200^Z, 125^Y, 125^X$ nm
**Training Dataset**	
Dataset size	2M synthetic samples
Number of beads	$b \in \{1 \longrightarrow 5\}$
Bead size	FWHM $\in \{100, 200, 300, 400\} nm$
Mode distribution(s)	$\mathcal{D} \in$ {Single, Bimodal, Powerlaw, Dirichlet}
Mode weighting (Z_n^m)	Uniform $n \in \{2 \longrightarrow 4\}$ & linear decay $n \in \{4 \longrightarrow 10\}$ *w.r.t.* m
Magnitude range	$a \in \{0 \longrightarrow 0.3\}\mu$m $\Longleftrightarrow a \in \{0 \longrightarrow 5\}\lambda$
Image size	$64^Z, 64^Y, 64^X$ voxels $\Longleftrightarrow 12.8^Z, 8^Y, 8^X$ μm
**Hyperparameters**	
Batch size	4096
Loss	MSE
Optimizer	AdamW [43]
Layerwise decay	LAMB [44]
Momentum β_1	0.9
Momentum β_2	0.99
Initial learning rate (LR)	0.0001
Weight decay	0.001
**Training schedulers**	
Total epochs	500
Warmup epochs	25
Warmup scheduler	Linear
Learning rate decay	Cosine [45]
